# Selective Sequential Depolymerization of Mixed Plastics Mediated by Photothermal Conversion

**DOI:** 10.1002/anie.2075380

**Published:** 2026-07-13

**Authors:** Yoon‐Jung Jang, Angela Milo, Deepika Shingwekar, Hanning Jiang, Erin E. Stache

**Affiliations:** ^1^ Department of Chemistry Princeton University Princeton New Jersey USA

**Keywords:** carbon black, chemical recycling, photothermal depolymerization, poly(ethylene terephthalate), polylactide, polystyrene

## Abstract

Chemical recycling of plastics into monomers is a promising strategy to achieve a circular economy. However, selective depolymerization methods for mixed plastics are still underdeveloped. Herein, we report a selective and sequential depolymerization strategy for mixed plastics, including poly(*L*‐lactide) (PLLA), polystyrene (PS), and poly(ethylene terephthalate) (PET), using photothermal conversion. We were able to selectively depolymerize PLLA into *L*‐lactide in the presence of PS and PET. Then, PS was selectively depolymerized to styrene, followed by the depolymerization of PET into its monomer. Our protocol was carried out in one pot without any additional purification of the unreacted plastics at each stage. This method was successfully applied to mixtures of post‐consumer waste plastic.

## Graphical abstract

This work provides a selective sequential depolymerization strategy for mixed plastic waste. The method enables depolymerization of poly(*L*‐lactide), polystyrene, and poly(ethylene terephthalate), including their post‐consumer plastics. Without presorting, monomer separation is achieved by leveraging the distinct chemical reactivities of each polymer in one pot.

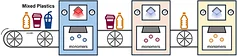

## 1 Introduction

The accumulation of plastic waste has raised significant environmental concerns. By 2015, 6300 million tons of plastic waste had been produced, of which less than 10% was recycled [1]. For example, polystyrene (PS), one of the standard plastics used in packaging and household items, has a recycling rate of less than 1% [2, 3, 4]. Poly(ethylene terephthalate) (PET), despite its widespread use in bottles, textiles, and food containers, has a recycling rate below 30% [3, 5]. Poly(*L*‐lactide) (PLLA) has emerged as the most prevalent sustainable alternative to petroleum‐based plastics due to its renewability of feedstock and degradability, currently achieving a global recycling rate of 2% [6, 7]. Approximately 79% of plastics have been deposited into landfills or mismanaged, leading to environmental leakage. The plastic debris causes environmental harm through fatal ingestion and entanglement in wildlife, as well as the generation of microplastics [8, 9, 10, 11]. Thus, effective plastic recycling strategies are essential to decrease waste generation and circularize the value of materials.

**FIGURE 1:**
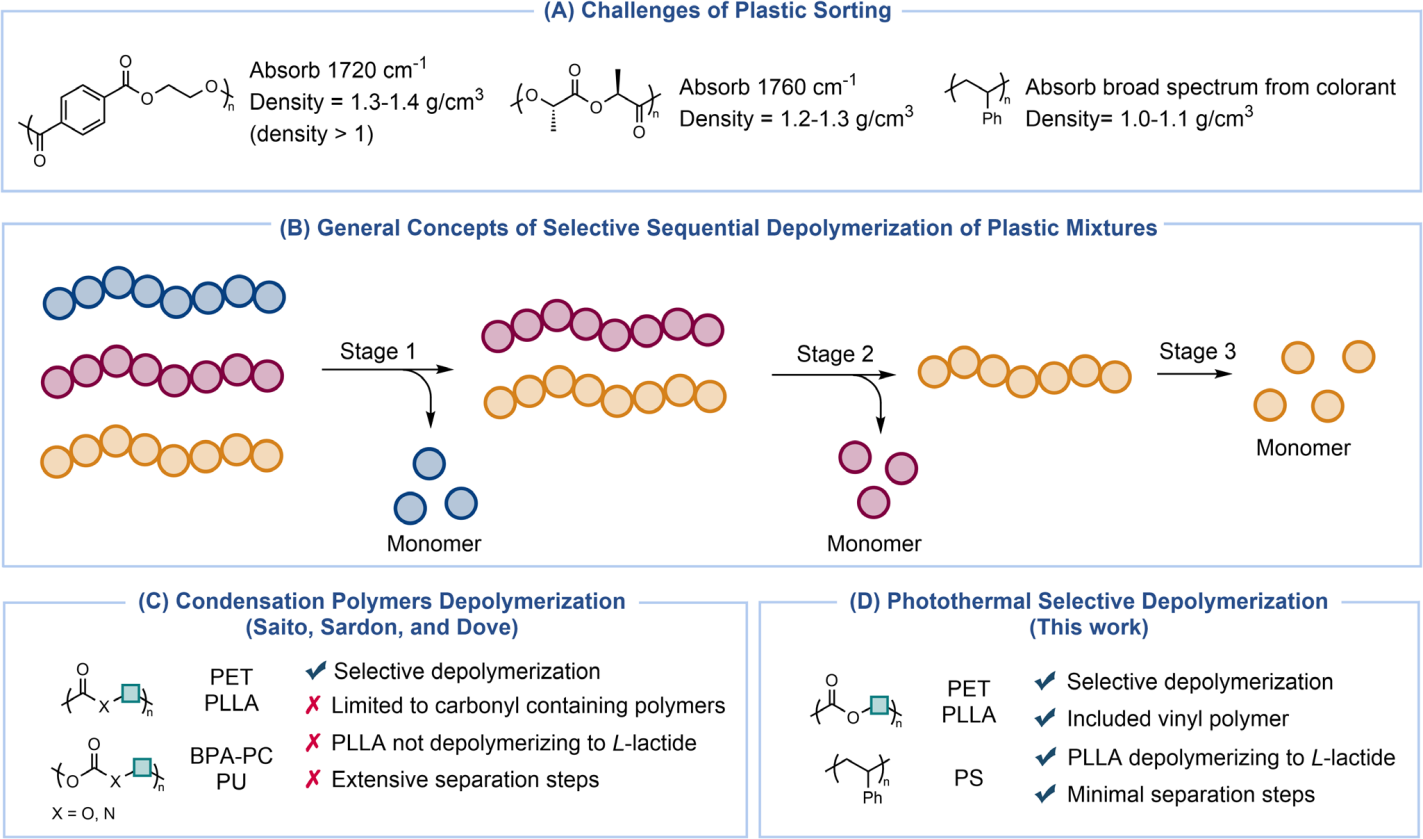
Selective depolymerization of plastic waste mixtures. (A) Challenges in plastic sorting for PET, PLLA, and PS [43, 44, 45, 46]. (B) Schematic diagram of selective sequential depolymerization of plastic mixtures. (C) Previous works for the selective depolymerization of mixed plastics. BPA‐PC is bisphenol‐A polycarbonate, and PU is polyurethane. (D) Selective photothermal depolymerization of mixed plastics.

Mechanical recycling is a predominant method of recycling plastic waste. In mechanical recycling, plastic mixtures are presorted using techniques, such as infrared (IR) spectroscopy and density separation [3, 12, 13, 14, 15]. However, presorting systems face challenges associated with similar identifying factors, such as overlapping IR peaks and similar densities (Figure 1A) [16]. Common colorants and dyes in commodity polymers, such as carbon black, further complicate separation due to their IR absorption, leading to the accidental inclusion of immiscible polymers [17, 18, 19]. Moreover, the presence of copolymers and multi‐layered plastics further complicates this, resulting in significantly diminished mechanical properties due to phase separation [12, 14, 15, 20]. Although chemical recycling can circumvent this downcycling issue by resynthesizing new plastics from recycled monomers, ineffective presorting still reduces the overall efficiency of the process [21]. Therefore, developing advanced chemical recycling methods for mixed plastic waste is desirable.

Selective sequential chemical recycling is an ideal strategy that depolymerizes plastic mixtures into their respective monomers at each stage, without presorting steps (Figure 1B). While most chemical recycling methods target single plastics, only limited examples demonstrate selective chemical recycling of mixed plastics. Pioneering works from the Sardon, Dove, and Saito groups designed selective depolymerization reactions by exploiting the energetic differences in the depolymerization of each polymer (Figure 1C) [22, 23, 24, 25, 26]. However, previous studies are limited to carbonyl‐containing polymers. Although polystyrene accounts for a third of landfill waste worldwide, it is excluded from the selective chemical recycling of mixed plastics. This is due to the extremely high temperatures (>400 °C) required for polystyrene degradation [4]. Furthermore, prior studies on mixed plastics used glycolysis or methanolysis, which depolymerized poly(*L*‐lactide) (PLLA) into lactic acid or alkyl lactates rather than regenerating the *L*‐lactide monomer [22, 25, 26]. These products require additional processing to afford lactide, accounting for 30% of the energy used in PLLA production in life cycle analyses [27]. Therefore, the development of chemical recycling strategies for mixed plastics, including diverse polymers beyond carbonyl classes, that regenerate desired monomers is needed.

Recently, photothermal conversion has attracted increasing attention as an efficient strategy for chemical recycling to monomers [28, 29, 30, 31, 32, 33, 34, 35, 36]. Photothermal conversion refers to the conversion of light into heat at the surface of photothermal agents (PTAs) upon irradiation, driven by non‐radiative decay processes such as vibrational relaxation [37, 38, 39, 40]. Since the bulk reaction mixture remains at a relatively low temperature compared to the elevated surface temperature of PTA (Δ*T ∝ r‐^1^)*, photothermal reactions can potentially yield products with high selectivity by minimizing undesirable side reactions [35, 41, 42]. Our group recently reported the depolymerization of black polystyrene by utilizing the photothermal conversion of black pigments already present as plastic additives [32].

By leveraging the tunability of photothermal conversion through light intensity and reagent addition, we report a selective, sequential depolymerization strategy for mixed plastics, including PLLA, PET, and PS (Figure 1D). PET and PLLA are difficult to sort due to their similar IR absorptions (around 1700 cm^−1^) (Figure 1A) [43, 45]. It was reported that up to 14% of PLLA can remain after IR‐based spectroscopic separation, which negatively impacts the recycling process [47]. Since PS is transparent, it is often colored with additives such as carbon black. The IR absorption of its pigments complicates the separation of PS from PET and PLLA [44]. Their densities are higher than that of water, making the traditional water‐based float‐sink process ineffective (Figure 1A) [48, 49, 50]. Our approach enabled the selective depolymerization of mixtures of PLLA, PS, and PET into their monomers in sequence. This strategy was also effective for post‐consumer plastics on a large scale (2 g), thereby paving the way for selective and sequential chemical recycling of mixed post‐consumer plastic waste.

## 2 Results and Discussions

**FIGURE 2:**
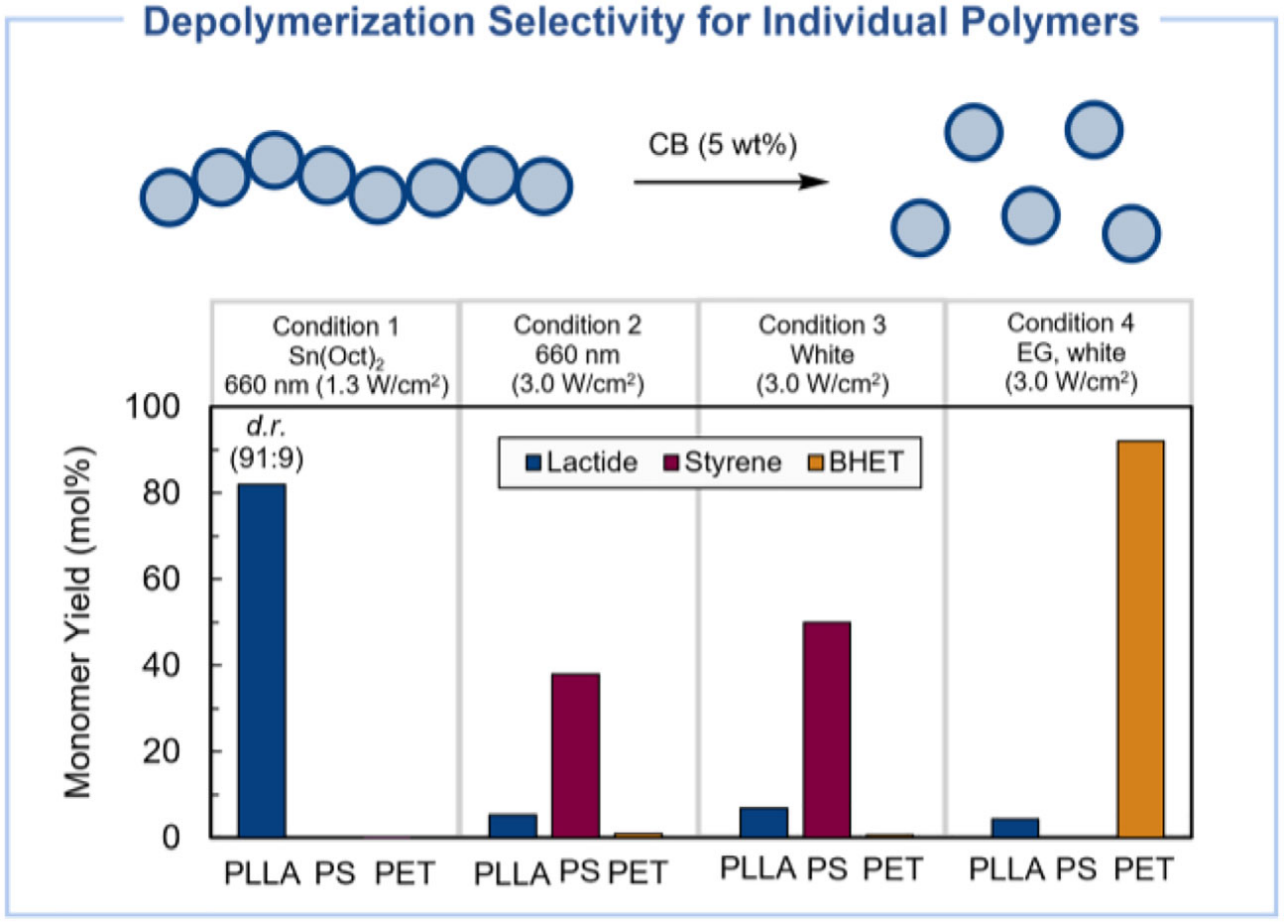
Photothermal depolymerization of PLLA, PS, and PET single‐plastic system. Lactide, styrene, and bis(2‐hydroxyethyl) terephthalate (BHET) are depolymerization products of PLLA, PS, and PET, respectively. Individual polymer powder (not plastic mixture) was mixed with carbon black (CB) by vortexing. Condition 1 is using 7 wt% of Sn(Oct)_2_ under static vacuum for 30 min. Condition 2 is under static vacuum for 20 min. Condition 3 is under static vacuum for 20 min. Condition 4 is using ethylene glycol (EG, 560 wt%) under and air for 30 min.

We began our studies by identifying photothermal depolymerization conditions that enabled the depolymerization of PLLA, PS, and PET (Figure 2). There are only a limited number of reports on the chemical recycling of PLLA to *L*‐lactide [22, 38, 39, 40, 41]. This depolymerization often suffers from side reactivity, including epimerization at elevated temperatures. We depolymerized PLLA using carbon black as a PTA under irradiation. After irradiation with a 1.3 W/cm^2^ 660 nm LED, a mixture of PLLA and carbon black yielded 1% lactide (Table S2). Catalyst screening revealed that tin (II) 2‐ethylhexanoate (Sn(Oct)_2_) afforded the highest efficiency, producing lactide in 82% yield. The Sn(Oct)_2_ catalyst also gave high stereoselectivity, generating a diastereomeric ratio of 91:9 (Figure 2, Condition 1). Considering the 0.96 ratio of the *iii* configuration (0.02 of *iis* and 0.02 of *isi*) from the initial polymer as determined by homonuclear decoupled ^1^H NMR spectroscopy, most of the stereogenic centers were maintained without significant epimerization during depolymerization (Figure S12). This high *L*‐lactide yield was comparable to previous works at elevated temperatures (140–210 °C), where PLLA depolymerization was proposed to predominantly involve a chain‐end backbiting mechanism [51]. These results suggested efficient photothermal depolymerization of PLLA toward *L*‐lactide under solvent‐free and mild visible light irradiation conditions [27, 51, 52, 53, 54].

Under the optimized conditions used for PLLA (irradiation using 1.3 W/cm^2^, 660 nm), a near quantitative amount of polystyrene was recovered, and less than 1% of styrene was obtained. The *M*_n_ of leftover PS was 59.8 kg/mol (*Đ* = 2.71), which is close to that of the initial *M*_n_ of 60.6 kg/mol (*Đ* = 2.76, Figure S11), suggesting that polystyrene was not reactive under these conditions (Figure 2, condition 1). When PET was subjected to the same conditions, almost negligible amounts of PET decomposition were observed. These data demonstrate that selective depolymerization under 1.3 W/cm^2^ light only occurred for PLLA. This is likely because activation energy for PLLA depolymerization (111 kJ/mol) in the presence of Sn(Oct)_2_ is lower than that for PS (221 kJ/mol) and PET (242 kJ/mol) [51, 55, 56, 57].

Since light intensity determines the number of incident photons, which in turn influences the number of excitation‐relaxation events, it strongly affects the rate of heat decay and thereby the energy available to effect depolymerization. When the intensity increased to 3.0 W/cm^2^, styrene was produced in 38% yield (Figure 2, condition 2). Under 6000 K white irradiation at the same intensity, the yield of styrene further increased to 50%, demonstrating successful polystyrene depolymerization (Figure 2, condition 3). This is likely due to the broader emission spectrum of the 6000 K white LED compared to the 660 nm LED (Figure S2). It has been reported that the mechanism of polystyrene photothermal depolymerization involves random chain scission followed by chain‐end unzipping [32]. However, when subjected to the same light conditions, the yield of lactide decreased due to the formation of acetaldehyde as a side product. For PET depolymerization under the same conditions, no significant BHET was detected. When ethylene glycol was added as a nucleophile under the same light irradiation, PET was depolymerized to BHET with 92% yield (Figure 2, Condition 4) [58, 59, 60]. These data demonstrate that light intensity and the presence of ethylene glycol are key components to the selective sequential depolymerization of PLLA, PS, and PET.

**FIGURE 3:**
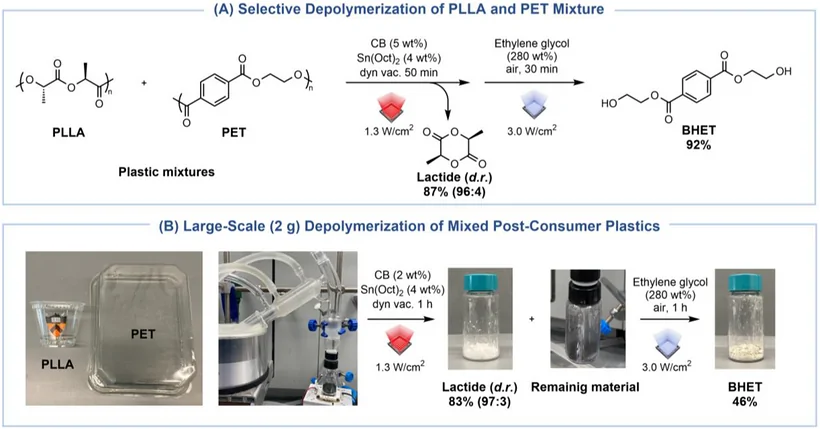
(A) Selective photothermal depolymerization of PLLA and PET mixture. (B) Large‐scale (2 g) depolymerization of a post‐consumer PLLA (1 g) and PET (1 g) mixture. Plastic powder mixtures were physically mixed with CB by vortexing.

We chose PLLA and PET for the selective depolymerization of two plastic mixtures. PET is one of the most mechanically recyclable plastics [3, 5]. However, PLLA can often act as a contaminant with current sorting systems in the recycled PET [50, 61, 62, 63, 64]. The impact strength and tensile properties of PET were significantly decreased by contamination with just 2% concentration of PLLA in PET [64]. Gratifyingly, we found that in a mixture of PET, PLLA, CB, and Sn(Oct)_2_, PLLA was selectively depolymerized back to *L*‐lactide (87% of yield, *d.r*. = 96:4) using 660 nm LED (1.3 W/cm^2^) and simultaneously separated from the reaction mixture using a distillation setup under dynamic vacuum (Figure 3A). Without any separation step, ethylene glycol was added to the leftover reaction vial to depolymerize PET to BHET, yielding 92% with a 6000 K white LED (3.0 W/cm^2^). These results show that selective depolymerization is highly efficient in PLLA‐PET plastic mixtures. To the best of our knowledge, this is the first report of selective sequential depolymerization of PLLA to *L*‐lactide in plastic mixtures.

Excitingly, we were able to perform the large‐scale (2 g total) depolymerization of post‐consumer mixed plastics containing PLLA and PET (Figure 3B). PLLA plastic cups and PET salad containers were mixed with Sn(Oct)_2_ and CB. After only 1 h of irradiation with a 660 nm LED (1.3 W/cm^2^), *L*‐lactide was obtained in 83% yield (*d.r*. = 97:3). Without additional purification of unreacted PET, the second step produced BHET with a 46% yield after recrystallization from methanol. While the yield was lower, likely due to a lower surface‐to‐volume ratio limiting light penetration and heat distribution, these results suggest the potential for scalability of this system for real plastics.

**FIGURE 4:**
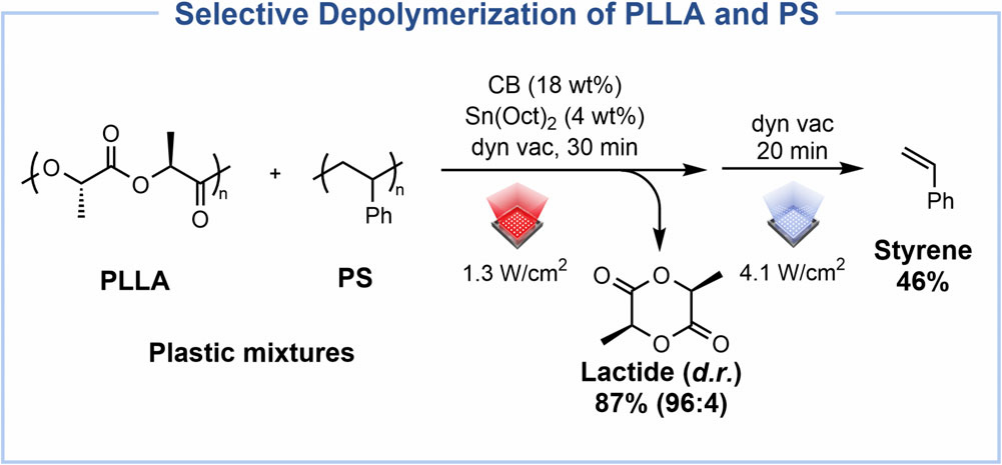
Photothermal selective depolymerization of PLLA and PS powder mixture.

We also performed the depolymerization of PLLA and PS mixture. In the first step, PLLA afforded 87% yield of *L*‐lactide with high stereocenter retention (*d.r*. = 96:4) under dynamic vacuum upon irradiation at 1.3 W/cm^2^ (Figure 4). Then, we switched to a more intense light source (4.1 W/cm^2^) for the second step, yielding 46% styrene within 20 min. Decreasing the light intensity and carbon black loading reduced styrene yield (Table S8). These data demonstrate the versatility of this system across different plastic mixtures.

**FIGURE 5:**
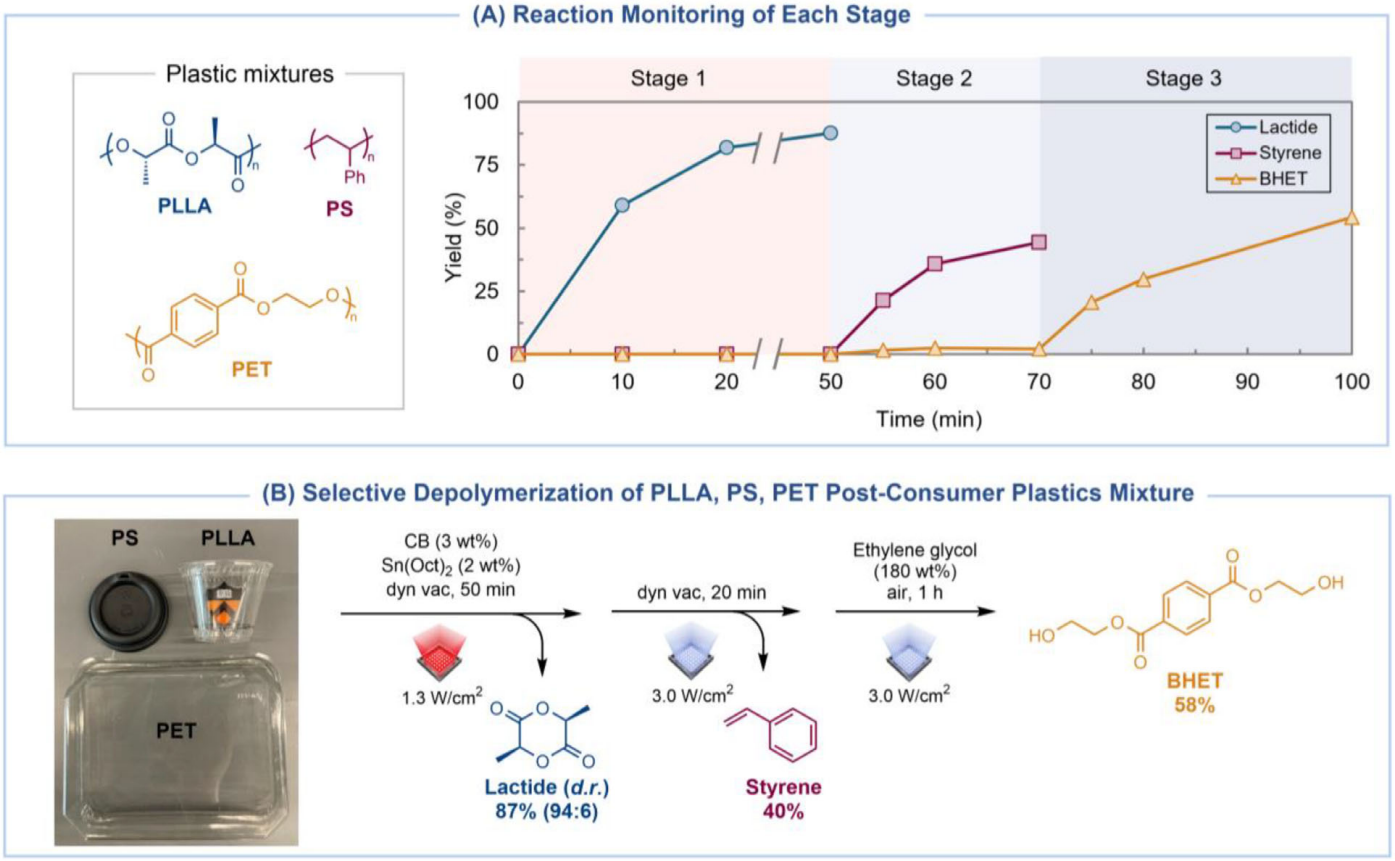
Selective photothermal depolymerization of PLLA, PS, and PET mixture. (A) Reaction monitoring of each stage using commercial polymer powders. (B) Selective depolymerization of post‐consumer plastic mixture powders. The yields are after purity correction of post‐consumer plastics. Plastic powder mixtures were physically mixed with CB by vortexing.

Encouraged by the success of selective depolymerization in two plastic systems, we applied this strategy to three plastics: PLLA, PS, and PET. In a mixed plastic system, we selectively depolymerized PLLA to *L*‐lactide in the first step, providing a similar yield to that achieved in a single plastic system. (Figure 5). We also monitored the yield of styrene and BHET, which can be generated from the depolymerization of PS and PET, respectively. Throughout the reaction, only a negligible amount of either styrene or BHET was observed in ^1^H NMR spectroscopy. These data show that the first step was selective only for PLLA depolymerization in the presence of PS and PET.

Without purifying the unreacted polymer mixture in the reaction setup, we performed the second step by switching to a more intense LED (3.0 W/cm^2^) to depolymerize PS to styrene. Under these conditions, 44% of styrene was generated after 20 min of light irradiation. The styrene yield was not significantly different from that in individual PS depolymerization (50%), demonstrating successful PS depolymerization in this mixed plastic system. We also monitored PET decomposition in the second step and observed 19% of terephthalic acid and 2% of BHET. These results show that styrene can be separated from the mixtures with minor PET decomposition.

As the third step, ethylene glycol was added to the leftover reaction mixture from the previous step, without purification. The depolymerization of PET produced BHET with a 54% yield after 30 min of irradiation. The yield of BHET was lower than that from individual PET depolymerization, presumably due to the formation of side products, such as terephthalic acid, acetaldehyde, and benzoic acid in the second step. Overall, these data highlight the feasibility and efficiency of achieving selective, simple depolymerization across three plastic systems.

We also demonstrated the feasibility of this sequential, selective depolymerization strategy on post‐consumer materials (Figure 5B). As black post‐consumer PS is difficult to separate from IR separation, we used a black PS coffee cup lid, a PLLA cup, and a PET salad container. Without separating black pigments in PS, the first step yielded 87% *L*‐lactide (*d.r*. = 94:6). In the second and third steps, 40% and 58% of styrene and BHET were generated, respectively. This method represents a promising approach for chemically recycling mixed plastics, given current challenges in plastic separation.

## 3 Conclusions

We report a selective and sequential depolymerization strategy for mixed plastics in one pot using photothermal conversion. From depolymerization studies of individual PLLA, PS, and PET, we found that light intensity modulation and the addition of ethylene glycol are key determinants of selectivity. In a mixed plastic system, we selectively depolymerized PLLA to *L*‐lactide in the first step in the presence of PET without reducing yield. Notably, for PLLA, *L*‐lactate stereogenic centers were largely maintained under these conditions. In the second step, without purifying the unreacted PET, it was also depolymerized to BHET in one pot with near‐quantitative yield. This system was applied to large‐scale (2 g) reactions of post‐consumer plastics. Moreover, this strategy was successfully extended to three plastic mixtures including PLLA, PS, and PET. This work demonstrates a robust strategy that enhances the practicality and feasibility of chemical recycling of mixed plastics providing a potential pathway toward more complex, real‐world plastic mixtures.

## Author Contributions

The manuscript was written with contributions from all authors. All authors have approved the final version of the manuscript.

## Conflicts of Interest

The authors declare no conflicts of interest.

## Supporting information



The supporting information includes experimental methods and data.
**Supporting File**: anie73607‐sup‐0001‐SuppMat.pdf.

## Data Availability

The data that support the findings of this study are available in the supplementary material of this article.
