# Supplementary material for: Selective Sequential Depolymerization of Mixed Plastics Mediated by Photothermal Conversion
Source: Angew Chem Int Ed Engl. 2026 Jul 13;65(36):e2075380. doi: 10.1002/anie.2075380 (PMC13528544; doi:10.1002/anie.2075380)
Supplement: Supplementary file 1 [file ANIE-65-e2075380-s001.pdf]

Supporting Information

Selective Sequential Depolymerization of Mixed Plastics Mediated by Photothermal Conversion

Yoon-Jung Jang[1], Angela Milo[1], Deepika Shingwekar[1], Hanning Jiang[1], and Erin E. Stache[1]*

[1]Department of Chemistry, Princeton University; Princeton, New Jersey, 08544, USA

Materials

Polystyrene (PS), 1,3,5-trimethoxybenzene, and $Sn(Oct)_2$ were purchased from Sigma-Aldrich and used as received. Poly(*L*-lactide) (PLLA) was purchased from Magerial and used as received. Poly(ethylene terephthalate) (PET) was obtained from a salad container from a grocery store. Carbon black (acetylene, 100% compressed) was purchased from Alfa Aesar and used as received. Ethylene glycol was purchased from Thermo scientific and dried over molecular sieves prior to use. Dichloromethane was purchased from Fisher Scientific and used as is. *d*-Chloroform ($CDCl_3$), *d*-dimethylsulfoxide (DMSO-d_6) were purchased from Chembridge Isotope Laboratories Inc. and used as received. Methanol was purchased from Fisher Scientific and used as received. Post-consumer plastics were gathered from grocery stores and cafes. Photothermal depolymerization was carried out using a TX aluminum heatsink cooling fan equipped with Chanzon LED chips, powered through a HLG-120H-36A LED power supply (Mean Well). The illumination system was assembled manually from components purchased separately via Amazon and Mouser Electronics. Light intensity was determined using a PM100D power meter with an S425C thermal sensor head (2.6 cm of diameter, Thorlabs). Irradiation sources included a 660 nm LED (1.3, 3.0 W/cm²) and a 6000 K white LED (2.6, 3.0 W/cm²).

Characterization Methods

^{1}H NMR spectra were obtained from Bruker 400 and 500 MHz at room temperature, using $CDCl_3$ and dimethyl sulfoxide-d_6. Quantitative analysis was performed using 1,3,5-trimethoxybenzene as an internal standard.

Molar masses of polymers were obtained by size exclusion chromatography (SEC) on Agilent 1260 Infinity II system equipped with an Agilent 1260 Infinity II Isocratic Pump, 1260 Infinity II Refractive Index detector, two SDV linear M columns (8 × 300 mm, 3 μm), and one SDV guard column (8 × 50 mm, 3 μm). Tetrahydrofuran (VWR Chemicals BDH, 99%) containing butylated hydroxytoluene served as the mobile phase at a flow rate of 1 ml/min and temperature of 35 °C.

General Procedure for Grinding plastics

Commercial PS pellets (M_n = 60.6 kg/mol, $Đ$ = 2.76) from Sigma Aldrich, PLLA pellets (M_n = 71.2 kg/mol, $Đ$ = 1.94), and PET salad container, PLLA university cup, and black PS coffee cup lid were ground using a mini electronic coffee grinder (Secura, 110 V) to a fine powder.

General Procedure for photothermal depolymerization of poly(*L*-lactide) under static vacuum

To a 1-dram vial, poly(*L*-lactide) (40 mg), carbon black (1 wt%), and $Sn(Oct)_2$ (2.5 mol%) was added, vortexed, and mixed with a spatula. The vial was evacuated and flushed with nitrogen 3 times and left under a static vacuum (around 50 mTorr). The reaction vial was placed 2 mm above a 660 nm LED light (1.3 W/cm²). After 30 minutes of irradiation, the reaction vial was removed from the light and cooled to room temperature. The vial was further cooled in liquid nitrogen. *d*-Chloroform (1.5 mL) and air were added to equilibrate pressure. A stock solution of 1,3,5-trimethoxy benzene (0.58 mL, 0.095 M in $CDCl_3$) was added and aliquots were taken for NMR and SEC analysis.

Procedure for photothermal polylactide depolymerization under dynamic vacuum

To a 1-dram vial, poly(*L*-lactide) (40 mg), carbon black (1 wt%), and $Sn(Oct)_2$ (2.5 mol%) were added, mixed with a spatula, and vortexed. PTFE tape was wrapped around the neck of the vial. For the setup, the vial was attached to a short path condenser equipped with a round bottom receiving flask. The side of the vial was wrapped with glass wool and covered with foil, 3 mm from the bottom of the vial. The setup was evacuated and flushed with nitrogen 3 times and left under a dynamic vacuum. The receiving flask was placed in a dry ice and isopropyl alcohol bath, and the condenser was attached to an ice water pump. The vial was placed 2 mm above a 660 nm LED light (1.3 W/cm²) and irradiated for 30 minutes. After 30 minutes, the setup was filled with nitrogen. The round bottom flask was removed and $CDCl_3$ (1.5 mL) was added. The reaction vial was removed. A new vial was equipped, the glass connector was removed from the short path condenser, and the connector was washed with $CDCl_3$ (1 mL) 3 times. A different vial was equipped, and the connector was reattached to the short path condenser. The short path condenser was washed with $CDCl_3$ (1 mL) twice. A stock solution of 1,3,5-trimethoxy benzene (0.58 mL, 0.095 M in $CDCl_3$) was added to each vial and the round bottom flask and aliquots were taken for NMR and SEC analysis.

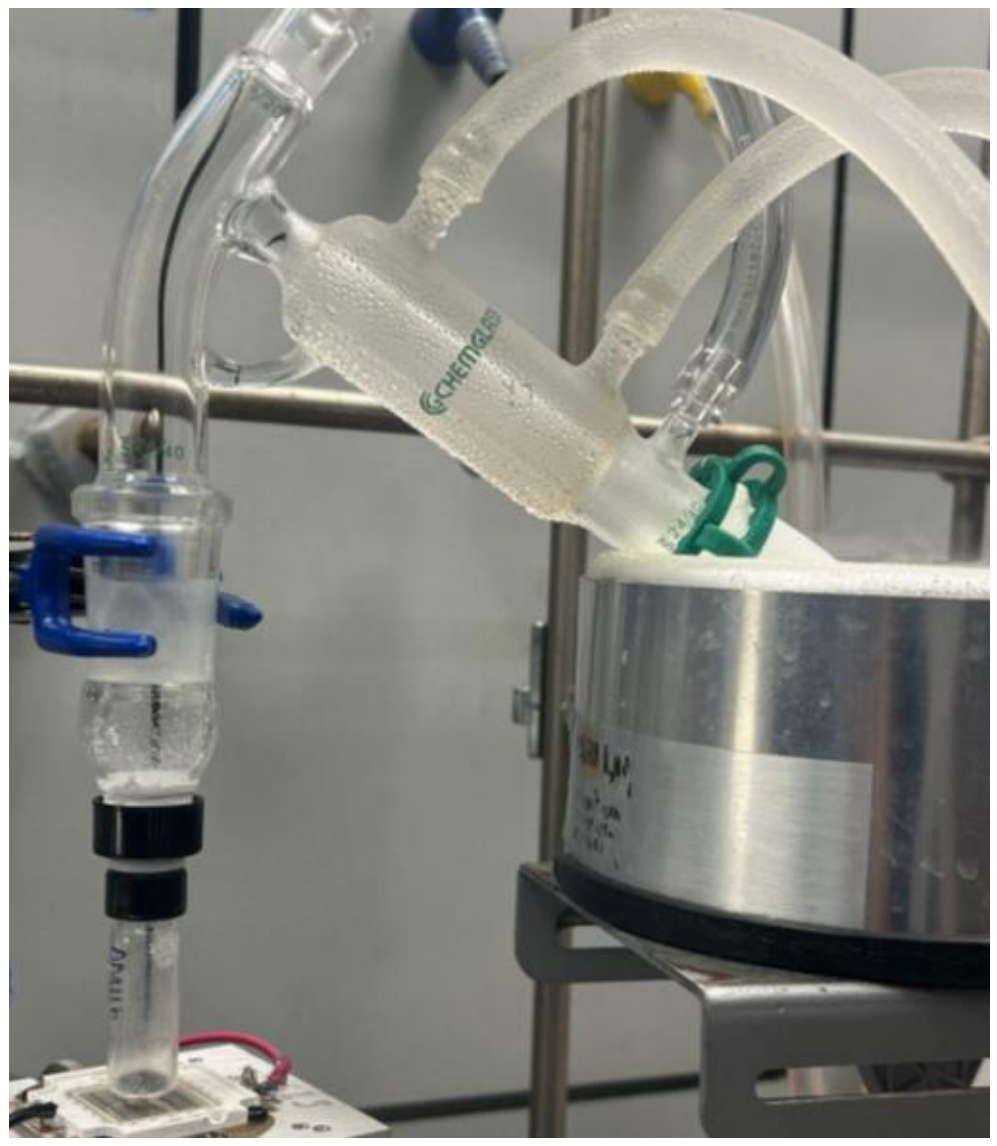

Figure S1. Reaction setup for depolymerization of PLLA under dynamic vacuum.

General Procedure for photothermal depolymerization of polystyrene under static vacuum

To a 1-dram vial, polystyrene (40 mg) and carbon black (1 wt%) were added, and vortexed. The vial was evacuated and flushed with nitrogen 3 times and left under a static vacuum (around 50 mTorr). The reaction vial was placed 2 mm above a 6000 K white LED (3.0 W/cm²). After 20 minutes of irradiation, the reaction vial was removed from the light and cooled to room temperature. The vial was cooled in liquid nitrogen. *d*-Chloroform (1.5 mL) and air were added to equilibrate pressure. A stock solution of 1,3,5-trimethoxy benzene (0.4 mL, 0.095 M in $CDCl_3$) was added and aliquots were taken for NMR and SEC analysis.

General Procedure for photothermal depolymerization of poly(ethylene terephthalate)

To a 1-dram vial, poly(ethylene terephthalate) (40 mg), carbon black (1 wt%), and ethylene glycol (0.2 mL) were added, and vortexed. The reaction vial was placed 2 mm above a 6000 K white LED (3.0 W/cm²). After 30 minutes of irradiation, the reaction vial was removed from the light and cooled to room temperature. A stock solution of 1,3,5-

trimethoxy benzene (0.22 mL, 0.095 M in *d*-dimethylsulfoxide (DMSO-d_6)) was added and aliquots were taken for NMR analysis.

General Procedure for photothermal depolymerization of poly(lactide and poly(ethylene terephthalate) mixture

To a 1-dram vial, poly(*L*-lactide) (40 mg), poly(ethylene terephthalate) (40 mg), carbon black (4 mg), and $Sn(Oct)_2$ (2.9 mg) were added, mixed with a spatula, and vortexed. PTFE tape was wrapped around the neck of the vial. For the setup, the vial was attached to a short path condenser equipped with a round bottom receiving flask. The setup was evacuated and flushed with nitrogen 3 times, and left under a dynamic vacuum. The vial was wrapped with glass wool and covered with foil, 3 mm from the bottom of the vial. The receiving flask was placed in a dry ice and isopropyl alcohol bath and the condenser was attached to an ice water pump. The vial was placed 2 mm above a 660 nm LED light (1.3 W/cm²) and irradiated for 50 mins. After 50 minutes, the setup was filled with nitrogen. The round bottom flask was removed and $CDCl_3$ (2 mL) was added. The reaction vial was removed. A new vial was equipped, the glass connector and short path were washed with $CDCl_3$ (1 mL) twice. A stock solution of 1,3,5-trimethoxy benzene (0.58 mL, 0.095 M in $CDCl_3$) was added to the each vial, and the round bottom flask and aliquots were taken for NMR analysis to obtain the yield of *L*-lactide. In the leftover reaction vial, ethylene glycol (0.2 mL) was added and the vial was equipped with a septum cap. The vial was vortexed, and placed 2 mm above a white LED (3.0 W/cm²), and irradiated for 30 minutes. After irradiation, the vial was removed from the light and cooled to room temperature. A stock solution of 1,3,5-trimethoxy benzene (0.22 mL, 0.095 M in $CDCl_3$), 0.72 mL of *d*-dimethylsulfoxide (DMSO-*d6*), and 0.5 ml of $CDCl_3$ were added and aliquots were taken for NMR analysis.

General Procedure for photothermal depolymerization of large-scale post-consumer polylactide and poly(ethylene terephthalate) mixtures

To a 20 mL vial, poly(*L*-lactide) (1 g), polyethylene terephthalate (1 g), carbon black (50 mg), and $Sn(Oct)_2$ (72 mg) were added, mixed with a spatula, and vortexed. PTFE tape was wrapped around the neck of the vial. For the setup, the vial was attached to a short path condenser equipped with a round bottom receiving flask. The setup was evacuated and flushed with nitrogen 3 times, and left under a dynamic vacuum. The vial was wrapped with glass wool and covered with foil, 3 mm from the bottom of the vial. The receiving flask was placed in a dry ice and isopropyl alcohol bath and the condenser was attached to an ice water pump. The vial was placed 2 mm above a 660 nm LED light (1.3 W/cm²) and irradiated for an hour. After irradiation, the setup was filled with nitrogen. The

round bottom flask was removed and $CDCl_3$ (2 mL) was added. *L*-Lactide was accumulated on the side of the reaction vial. Using spatula, white solid on the side wall was taken and transferred to the new vial. The white solid was dissolved in dichloromethane. The glass connector and the short path condenser were washed with dichloromethane. The solution was filtered through celite. The filtered solution was concentrated in vacuo and then dried overnight. The isolated mass was measured, and the product was analyzed by ^1H NMR spectroscopy.

In the leftover reaction vial, ethylene glycol (5 mL) was added and the vial was equipped with a septum cap. The vial was placed 2 mm above a white LED (3.0 W/cm^2) and irradiated for an hour. After irradiation, the vial was removed from the light and cooled to room temperature. Methanol was added to the vial and filtered through celite to remove carbon black and unreacted PET. The filtered solution was concentrated in vacuo and water was added to precipitate BHET. The solid was filtered, and washed with water, and dried overnight. The isolated mass was measured and the product was analyzed by ^1H NMR spectroscopy.

Procedure for photothermal depolymerization of mixed poly(*L*-lactide) and polystyrene under dynamic vacuum

To a 1-dram vial, poly(*L*-lactide) (40 mg), polystyrene (40 mg), carbon black (18 mg, 18.4 wt%), and $Sn(Oct)_2$ (2.9 mg, 2.5 mol%) were added, mixed with a spatula, and vortexed. PTFE tape was wrapped around the neck of the vial. For the setup, the vial was attached to a short path condenser equipped with a round bottom receiving flask. The setup was evacuated and flushed with nitrogen 3 times, and left under a dynamic vacuum. The vial was wrapped with glass wool and covered with foil, 3 mm from the bottom of the vial. The receiving flask was placed in a dry ice and isopropyl alcohol bath and the condenser was attached to an ice water pump. The vial was placed 2 mm above a 660 nm LED light (1.3 W/cm^2) and irradiated for 30 minutes. The setup was filled with nitrogen. The round bottom flask was removed and $CDCl_3$ (1.5 mL) was added. The reaction vial was removed. A new vial was equipped, the glass connector was removed from the short path condenser, and the connector was washed with $CDCl_3$ (1 mL) 3 times. A different vial was equipped, and the connector was reattached to the short path condenser. The short path condenser was washed with $CDCl_3$ (1 mL) twice. A stock solution of 1,3,5-trimethoxy benzene (0.58 mL, 0.095 M in $CDCl_3$) was added to the connector vial, short path vial, and the round bottom flask and aliquots were taken for NMR analysis.

The leftover reaction vial was attached to a new short path condenser and round bottom flask. The setup was evacuated and flushed with nitrogen 3 times, and left under a dynamic vacuum. The receiving flask was placed in a dry ice and isopropyl alcohol bath and the condenser was attached to an ice water pump. The vial was placed 1 mm above

a white LED light (14.0 W) and irradiated for 20 mins. The setup was filled with nitrogen and left undisturbed until the setup was room temperature. The round bottom flask was removed and $CDCl_3$ (1.5 mL) was added. The reaction vial was removed and $CDCl_3$ (1.5 mL) was added. A new vial was equipped and the connector and the short path condenser were washed with $CDCl_3$ (1 mL) 3 times. The short path condenser was washed with $CDCl_3$ (1 mL) twice. A stock solution of 1,3,5-trimethoxy benzene (0.4 mL, 0.095 M in $CDCl_3$) was added to the reaction vial, each vial (washed for short path and connector), and the round bottom flask and aliquots were taken for NMR analysis.

Procedure for photothermal depolymerization of mixed poly(*L*-lactide), polystyrene, and poly(ethylene terephthalate) under dynamic vacuum

To a 1-dram vial, poly(*L*-lactide) (40 mg), polystyrene (40 mg), and poly(ethylene terephthalate) (40 mg), carbon black (4 mg), and $Sn(Oct)_2$ (2.9 mg) were added, mixed with a spatula, and vortexed. PTFE tape was wrapped around the neck of the vial. For the setup, the vial was attached to a short path condenser equipped with a round bottom receiving flask. The setup was evacuated and flushed with nitrogen 3 times, and left under a dynamic vacuum. The vial was wrapped with glass wool and covered with foil, 3 mm from the bottom of the vial. The receiving flask was placed in a dry ice and isopropyl alcohol bath and the condenser was attached to an ice water pump. The vial was placed 2 mm above a 660 nm LED light (1.3 W/cm^2) and irradiated for 50 minutes. The setup was filled with nitrogen. The round bottom flask was removed and $CDCl_3$ (1.5 mL) was added. The reaction vial was removed. A new vial was equipped, and the glass connector and the connector were washed with $CDCl_3$ (1 mL) twice. A stock solution of 1,3,5-trimethoxy benzene (0.58 mL, 0.095 M in $CDCl_3$) was added to the vials, and the round bottom flask and aliquots were taken for NMR analysis.

The leftover reaction vial was attached to a new short path condenser and round bottom flask. The setup was evacuated and flushed with nitrogen 3 times, and left under a dynamic vacuum. The receiving flask was placed in a dry ice and isopropyl alcohol bath and the condenser was attached to an ice water pump. The vial was placed 1 mm above a 6000 K white LED (3.0 W/cm^2) and irradiated for 20 mins. After irradiation, the setup was filled with nitrogen and defrost to room temperature. The round bottom flask was removed and $CDCl_3$ (1.5 mL) was added. The reaction vial was removed. A new vial was equipped and the connector and short path condenser were washed with $CDCl_3$ (1 mL). A stock solution of 1,3,5-trimethoxy benzene (0.4 mL, 0.095 M in $CDCl_3$) was added to the vials, and the round bottom flask and aliquots were taken for NMR analysis.

Ethylene glycol (0.2 mL) was added to the leftover reaction vial. The vial was equipped with a septum cap, placed 1 mm above a 6000 K white LED (3.0 W/cm^2), and irradiated for 30 minutes. After irradiation, the vial was removed from the light and cooled

to room temperature. A stock solution of 1,3,5-trimethoxy benzene (0.22 mL, 0.095 M in $CDCl_3$), 0.72 mL of *d*-dimethylsulfoxide (DMSO-*d6*), and 0.5 ml of $CDCl_3$ were added and sonicated for 10 minutes. Aliquots were taken for NMR analysis.

Procedure for Bulk Temperature Measurements

CB (200 mg) was added to a 1-dram glass vial and sealed with a cap fitted with a Teflon septum. A small hole was pierced through the Teflon septum, and foil-wrapped thermocouple was inserted into the hole until the tip of the thermocouple was fully submerged in the solid contents of the vial. The vial was subsequently placed 0.2 mm above either a 660 nm LED (1.3 W/cm^2) or 6000 K white (3.0 W/cm^2), and irradiated for 20 minutes. The temperature measured by the thermocouple was recorded every 30 seconds for the first 5 minutes of irradiation, then every 60 seconds for the rest of the irradiation time. After the irradiation time finished, the thermocouple temperature was measured every 60 seconds for 5 minutes.

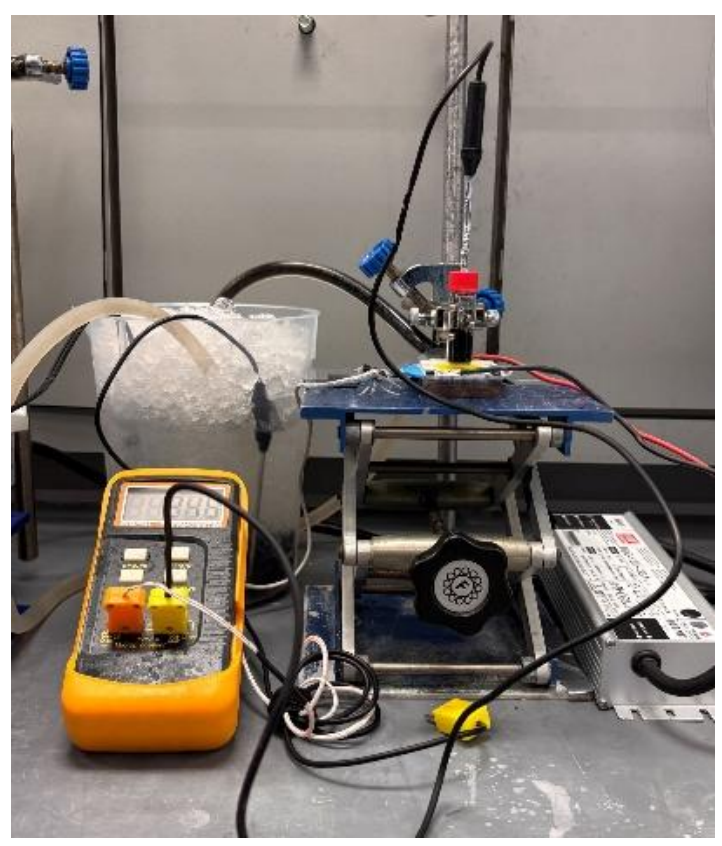

Procedure for Background Temperature Measurements

To measure the background temperature of the LED light chip, modifications were made to the bulk temperature measurement procedure. A 1-dram glass vial was sealed with a cap fitted with a teflon septum. A small hole was pierced through the teflon septum, and foil-wrapped thermocouple was inserted into the hole at a 2.5 cm depth, where the thermocouple tip was approximately 0.5 cm away from the bottom of the vial. Care was taken to ensure that the thermocouple was not touching the sides of the glass vial. The vial was subsequently placed 0.2 mm above either a 660 nm LED (1.3 W/cm^2) or 6000 K white (3.0 W/cm^2), and irradiated for 20 minutes. The temperature measured by the thermocouple was recorded every 30 seconds for the first 5 minutes of irradiation, then every 60 seconds for the rest of the irradiation time. After the irradiation time finished, the

thermocouple temperature was measured every 60 seconds for 5 minutes. The results are summarized below.

Procedure for Bulk Temperature Measurements with Ethylene Glycol

CB (10.5 mg) and ethylene glycol (1 mL, 17.9 mmol) were added to a 1-dram glass vial and sealed with a cap fitted with a Teflon septum. A small hole was pierced through the Teflon septum, and foil-wrapped thermocouple was inserted into the hole until the tip of the thermocouple was fully submerged in the solid contents of the vial. The vial was subsequently placed 0.2 mm above a 6000K white LED (3.0 W/cm^2) and irradiated for 30 minutes. The temperature measured by the thermocouple was recorded every 30 seconds for the first 5 minutes of irradiation, then every 60 seconds for the rest of the irradiation time. After the irradiation time finished, the thermocouple temperature was measured every 60 seconds for 5 minutes.

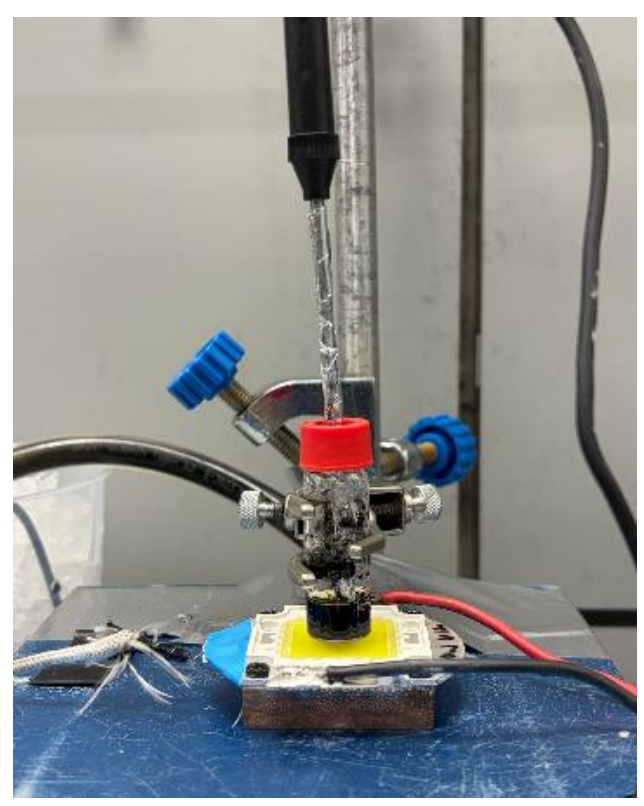

Procedure for Background Temperature Measurements with Ethylene Glycol

To measure the background temperature of the LED light chip, modifications were made to the bulk temperature measurement procedure. Ethylene glycol (1 mL) was added to 1-dram glass vial and was sealed with a cap fitted with a Teflon septum. A small hole was pierced through the Teflon septum, and foil-wrapped thermocouple was inserted into the hole at a 2.5 cm depth, where the thermocouple tip was approximately 0.5 cm away from the bottom of the vial. Care was taken to ensure that the thermocouple was not touching the sides of the glass vial. The vial was subsequently placed 0.2 mm above a 6000K white LED (3.0 W/cm^2) and irradiated for 30 minutes. The temperature measured by the thermocouple was recorded every 30 seconds for the first 5 minutes of irradiation, then every 60 seconds for the rest of the irradiation time. After the irradiation time finished, the thermocouple temperature was measured every 60 seconds for 5 minutes.

Procedure for Thermal PLLA Depolymerization

An sand bath was preheated for 30 minutes to 200 ºC, and left to equilibrate for an additional 30 minutes at 200 ºC. PLLA (40 mg, 0.555 mmol) and $SnOct_2$ (2.9 mg, 0.00716 mmol, 0.129 mol equiv) were added to a 1-dram vial with PTFE septa cap, then vortexed to mix well. The vial was subjected to three vacuum-N_2 refill cycles and was left under static vacuum after a fourth vacuum pull. The vial was inserted in the sand bath for a varied amount of time, with the sand bath temperature monitored by a thermocouple inserted at the same depth of the vial. After the reaction, the vial was immediately cooled using a dry-ice acetone bath, and $CDCl_3$ (1 mL) was added. A stock solution of TMB (0.2 mL of a 0.0276 M in $CDCl_3$) was added and aliquots were taken for NMR (0.2 mL reaction mixture was diluted with 0.2 mL $CDCl_3$) and SEC analysis. All reaction timepoints were run in duplicate.

Procedure for Thermal PS Depolymerization

A sand bath was preheated for 30 minutes to 400 ºC, and left to equilibrate for 30 minutes at 400 ºC. PS (40 mg, 0.348 mmol) was added to a 1-dram vial with PTFE septa cap, then vortexed to mix well. The vial was subjected to three vacuum-N_2 refill cycles and was left under static vacuum after a fourth vacuum pull. The vial was inserted in the sand bath for a varied amount of time, with the sand bath temperature monitored by a thermocouple inserted at the same depth of the vial. After the reaction, the vial was immediately cooled using a dry-ice acetone bath, and $CDCl_3$ (1 mL) was added. A stock solution of TMB (0.2 mL of a 0.0276 M in $CDCl_3$) was added and aliquots were taken for NMR (0.2 mL reaction mixture was diluted with 0.2 mL $CDCl_3$) and SEC analysis. All reaction timepoints were run in duplicate.

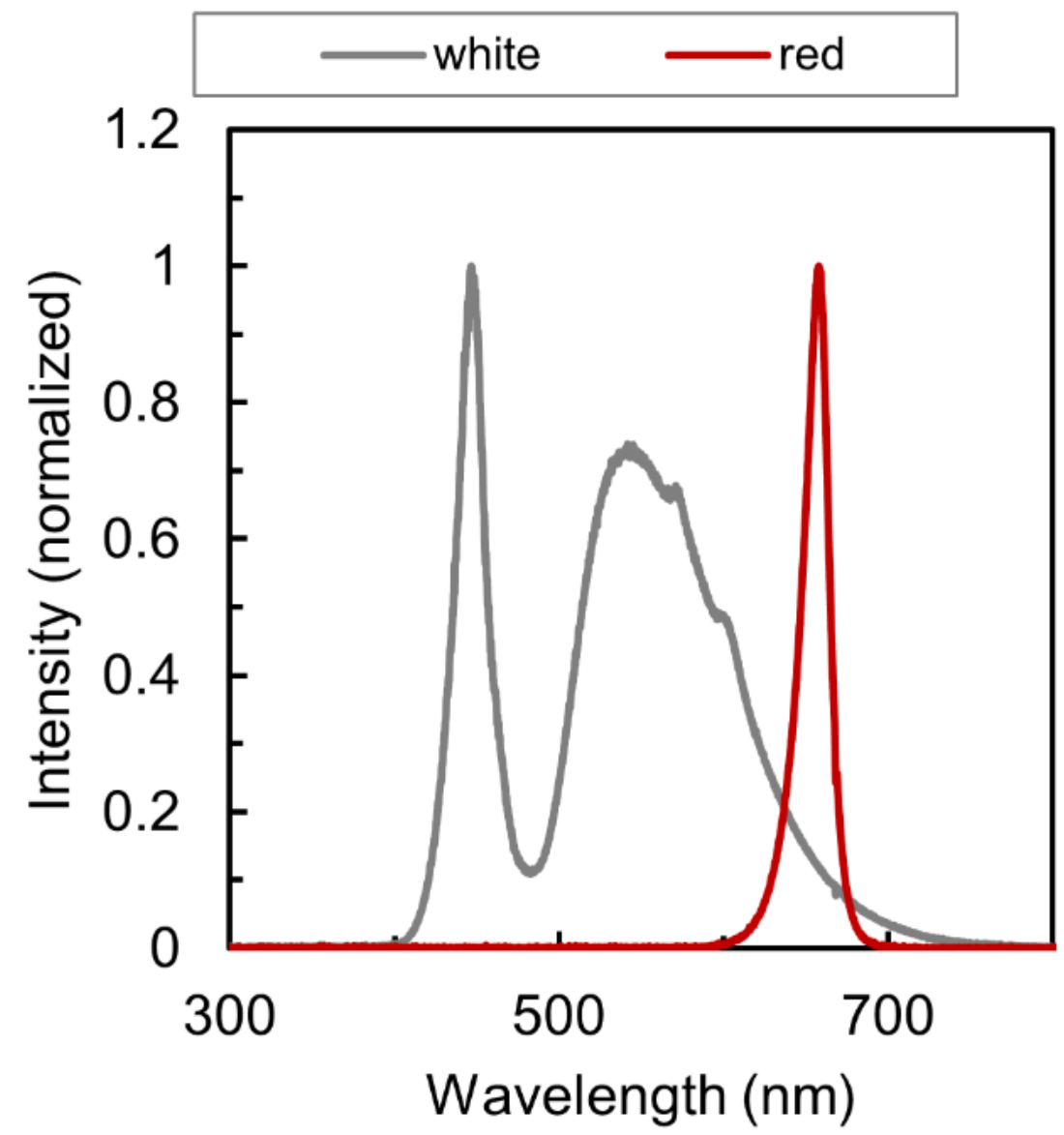

Figure S2. Emission spectra of 6000 K white and 660 nm LED.

Optimization for Photothermal PLLA Depolymerization

Table S1. Time Screening for Photothermal PLLA Depolymerization

Entry	time (min)	lactide (%)	*d.r.*	leftover PLA (%)	acetaldehyde (%)	mass recov. (%)
1	10	72.5	90:10	25.4	0.03	97.9
2	30	82.3	92:8	15.2	0.06	97.6
3	60	86.6	91:9	6.9	0.01	93.5

Depolymerization was performed at 40 mg scale of PLLA using 5 wt% of carbon black and a 660 nm LED light (1.3 W/cm²) under static vacuum

Table S2. Catalyst Screening for Photothermal PLLA Depolymerization

Entry	catalyst	lactide (%)	*d.r.*	leftover PLA (%)	acetaldehyde (%)	mass recov. (%)
1	$Sn(Oct)_2$	82.3	91:9	15.2	0.06	97.6
2	$Zn(OAc)_2$	60.1	86:4	31.4	0.18	91.7
3	$Zn(stea)_2$	55.5	83:7	47.6	0.10	103.2
4	$ZnCl_2$	54.5	70:30	23.1	0.37	78.0
5	ZnO	37.2	88:12	58.0	0.16	95.4
6	CaO	43.3	78:22	59.3	0.04	102.6
7	CaO[a]	40.6	72:28	59.5	0.05	100.2
8	MgO	9.2	72:28	94.6	0.04	103.8
9	MgO[a]	34.3	79:21	67.9	0.08	102.3
10	TiO_2	0	-	103.1	0.02	103.1
11	TiO_2[a]	0	-	100.5	0.05	100.6
12	$FeCl_3$	0	-	91.3	0.04	91.3
13	none	1.2	-	98.1	0.01	99.3

Depolymerization was performed at 40 mg scale of PLLA using a 660 nm LED light (1.3 W/cm²) for 30 minutes under static vacuum. [a]Performed at 80 mg scale of PLA for 2 hours.

Table S3. Carbon Black Loading Screening for PLLA Depolymerization

Entry	CB (wt%)	lactide (%)	*d.r.*	leftover PLA (%)	acetaldehyde (%)	mass recov. (%)
1	0.5	55.2	72:28	40.0	1.23	96.4
2	0.75	45.8	81:19	51.9	0.36	98.1
3	1	92.8	90:10	7.2	0.06	100.1
4	3	91.3	89:11	8.5	0.06	99.9

5	5	82.3	92:8	15.2	0.06	97.6
6	10	63.5	83:17	36.2	0.39	100.1

Depolymerization was performed at 40 mg scale of PLLA using a 660 nm LED light (1.3 W/cm²) under static vacuum for 30 minutes.

Table S4. Optimization of Catalyst Loading for Photothermal PLLA Depolymerization.

Entry	$Sn(Oct)_2$ (mol%)	lactide (%)	*d.r.*	leftover PLA (%)	acetaldehyde (%)	mass recov. (%)
1	0.5	68.4	90:10	27.5	0.02	95.9
2	1	87.6	90:10	9.2	0.05	96.9
3	2.5	82.3	92:8	15.2	0.06	97.6
4	5	84.2	88:12	8.3	0.05	92.6

Table S5. Comparison between Static and Dynamic Vacuum for PLLA Depolymerization.

Entry	Vacuum	lactide (%)	*d.r.*	leftover PLA (%)	acetaldehyde (%)	mass recov. (%)
1	Static Vacuum	82.3	92:8	15.2	0.06	97.6
2	Dynamic Vacuum	83.8	94:6	8.8	0	92.5

Table S6. Photothermal depolymerization of Polystyrene under Static Vacuum.

Entry	LED	Intensity (W/cm^2)	Styrene (wt%)	Dimer (wt%)	Trimer (wt%)	AMS (wt%)	Tol (wt%)	Selectivity (mol ratio)	PS (wt%)	Mass rec. (%)
1	660 nm	1.3	0.1	<0.1	<0.1	<0.1	<0.1	<0.1	>99	>99
2	660 nm	3.0	37.9	7.9	13.2	0.3	0.9	0.80	33.9	94.0
3	6000 K white	3.0	50.4	9.7	14.4	0.6	1.3	0.81	16.6	92.9

The reaction was performed under static vacuum. Each data point is an average of two runs. Entry 1 is a single experiment for 30 min. Reaction time for entry 2&3 is 20 min.

Table S7. Photothermal depolymerization of Poly(ethylene terephthalate) under Air.

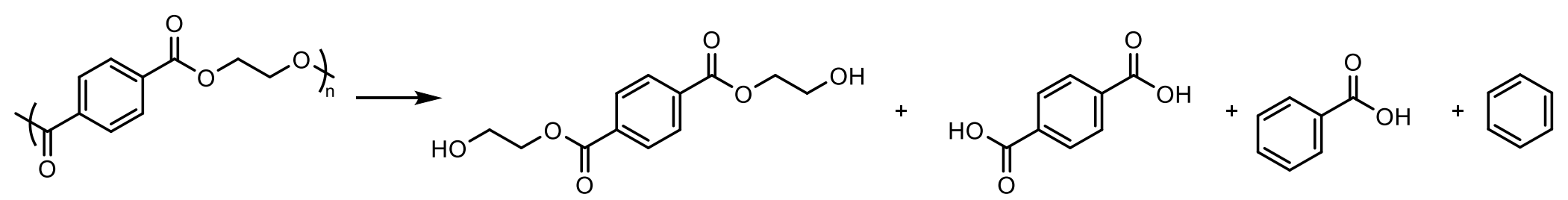

PET **BHET** **TPA** **BA** **Benzene**

Entry	Ethylene glycol	LED	Intensity (W/cm^2)	BHET (mol%)	TPA (mol%)	Acetaldehyde (mol%)	BA (mol%)	Benzene (mol%)
1	None	660 mm	1.3	<0.1	<0.1	<0.1	<0.1	<0.1
2[a]	None	660 mm	3.0	1	19.5	10.8	7.3	1.1
3[a]	None	6000 K white	3.0	<1	19.2	6.8	1.6	0.3
4	0.2 ml	660 nm	3.0	81.9	0.8	0.1	<0.1	<0.1
5	0.2 ml	6000 K white	3.0	91.3	9.9	0.5	<0.1	<0.1

[a]The reaction was performed under static vacuum to mimic the reaction conditions of PLLA and PS depolymerization.

Table S8. Optimization for Photothermal Depolymerization of PLLA and PS Mixture.

entry	CB (wt%)	1st step		2nd step
		lactide (%)	*d.r.*	styrene (%)
1	2.4	84.7	91:9	18.5
2	7	87.2	94:6	32.1
3	13	90.5	95:5	35.9
4	18.4	93.3	93:7	40.3
5	23.1	88.1	95:5	16.2
6[a]	18.4	87.4	96:4	46.4

Depolymerization of PLLA and PS was performed using a 660 nm LED light (1.3 W/cm^2) under dynamic vacuum for 30 minutes as the first step, and using a 6000 K white LED (2.6 W/cm^2) under dynamic vacuum for 20 minutes as the second step. The second step was performed using a 6000 K white LED (4.1 W/cm^2) under dynamic vacuum for 20 minutes.

Table S9. Optimization for Carbon Black Loading for PS Depolymerization in the PLLA, PS, and PET mixtures.

entry	CB (wt%)	1st step			2nd step		
		Time (min)	lactide (%)	*d.r.*	Intensity (W)	Time (min)	styrene (%)
1	0.3	30	60.4	92:8	2.6	20	0
2	1.6	30	88.3	96:4	2.6	20	4.1
3	3.2	30	85.2	96:4	2.6	20	39
9	3.2	30	87.9	96:4	3.0	20	44.6
4	4.7	30	91.0	96:4	2.6	20	39
5	9.1	30	92.9	96:4	2.6	20	19.5
6	13	30	90.0	95:5	2.6	20	12.6
7	25	30	73.9	94:6	2.6	20	10.5
8	33	30	70.5	91:9	2.6	20	0

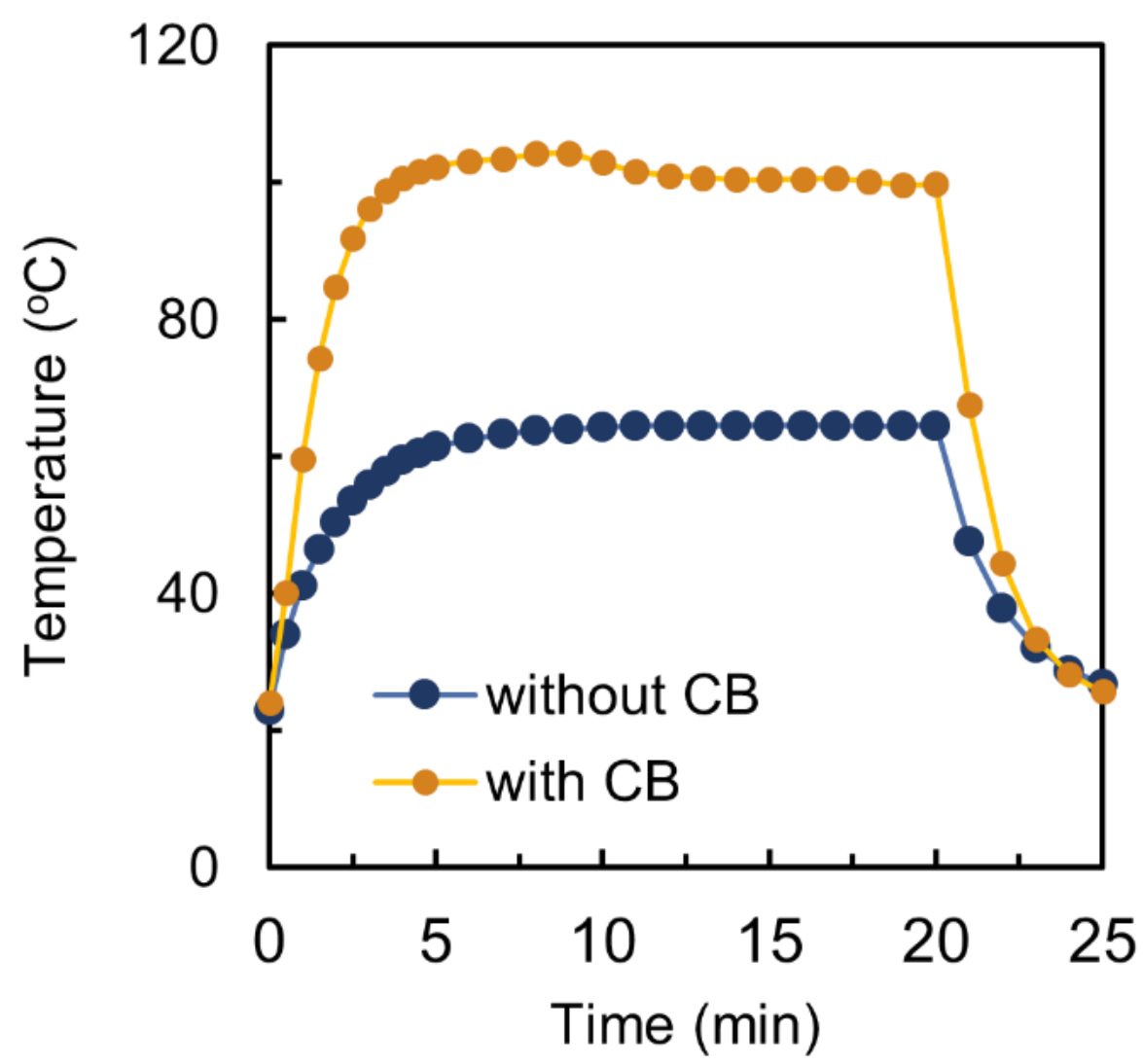

Figure S3. Measurement of the bulk temperature during the reaction upon irradiation with a 660 nm LED (1.3 W/cm^2).

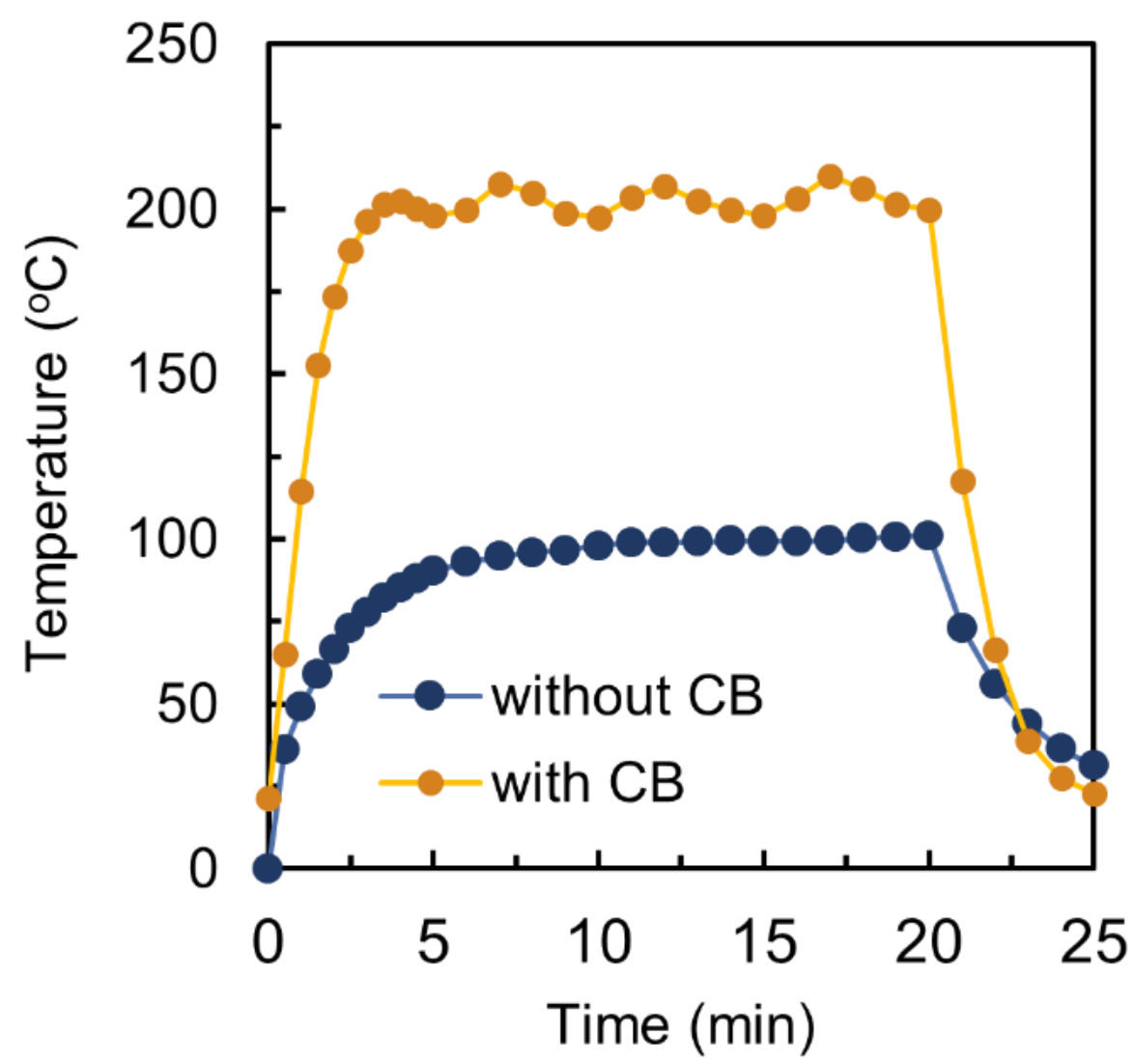

Figure S4. Measurement of the bulk temperature during the reaction upon irradiation with a 6000 K white LED (3.0 W/cm^2).

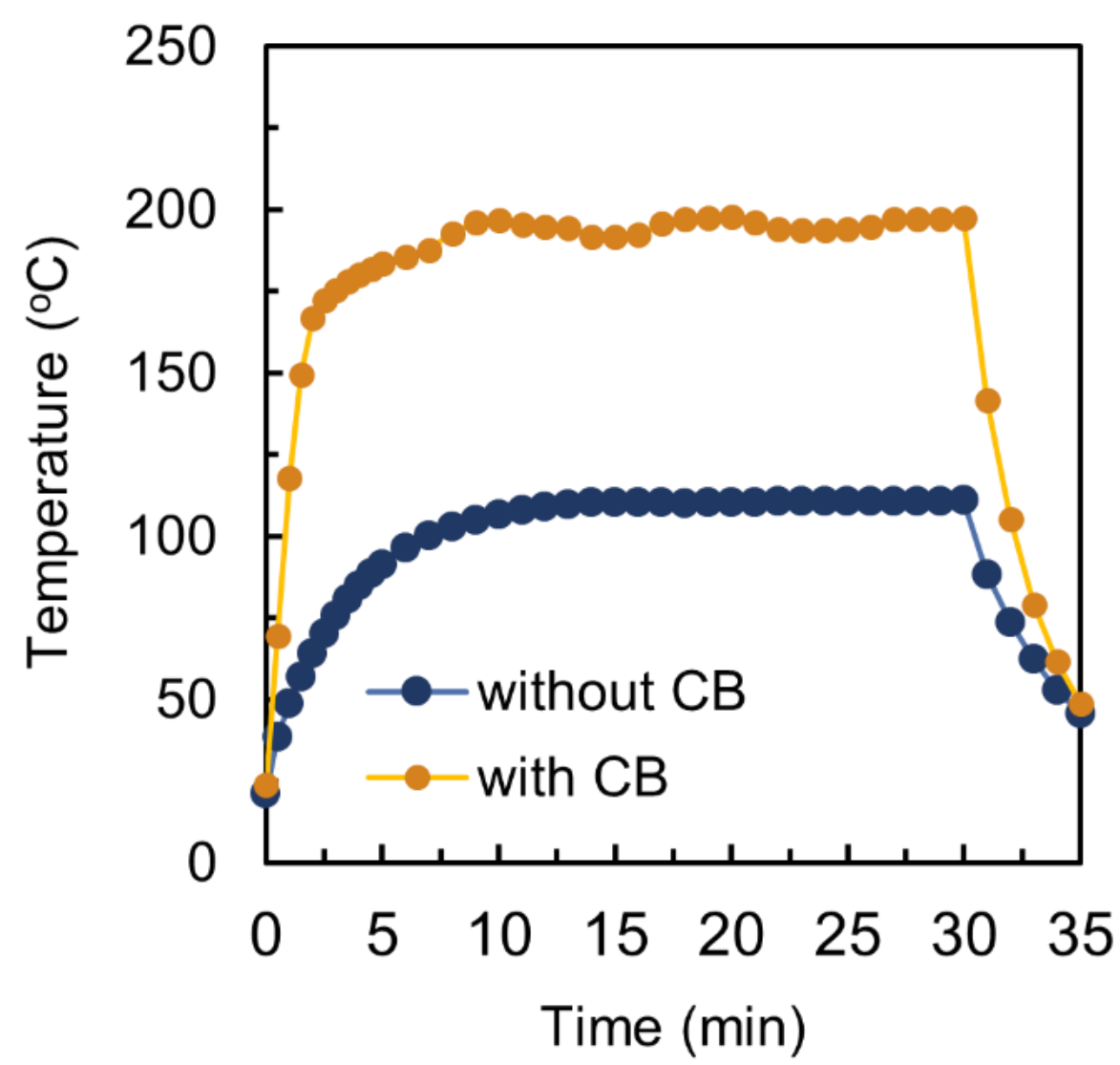

Figure S5. Measurement of the bulk temperature during the reaction upon irradiation with a 6000 K white LED (3.0 W/cm^2) in the presence of ethylene glycol.

Estimation of the local temperature[1-3]

The apparent photothermal temperatures have been estimated based on the Arrhenius equation. The rate constants ($k_{photothermal}$ and $k_{thermal}$) were obtained from the kinetic plots (Figure S6 – S9). *The estimated apparent temperature using 1.3 and 3.0 W/cm^2 LED were approximately 214 and 361 °C, respectively.*

$$k = Ae^{-E_a/RT}$$

$$lnk_1 - lnk_2 = -\frac{E_a}{R}(\frac{1}{T_1} - \frac{1}{T_2})$$

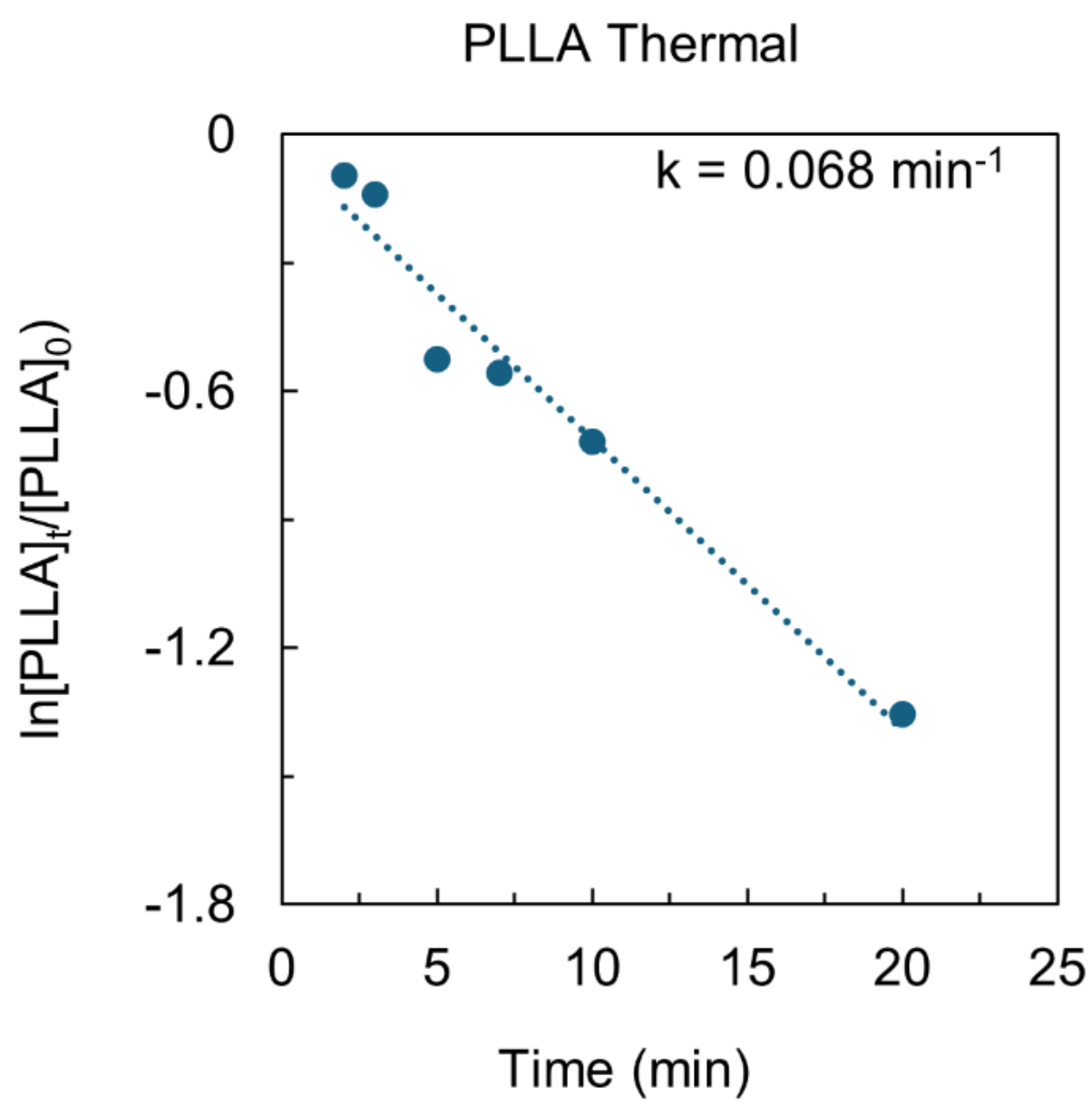

Figure S6. Kinetic plots of PLLA depolymerization at 200 °C in the presence of $Sn(Oct)_2$ (2.5 mol%).

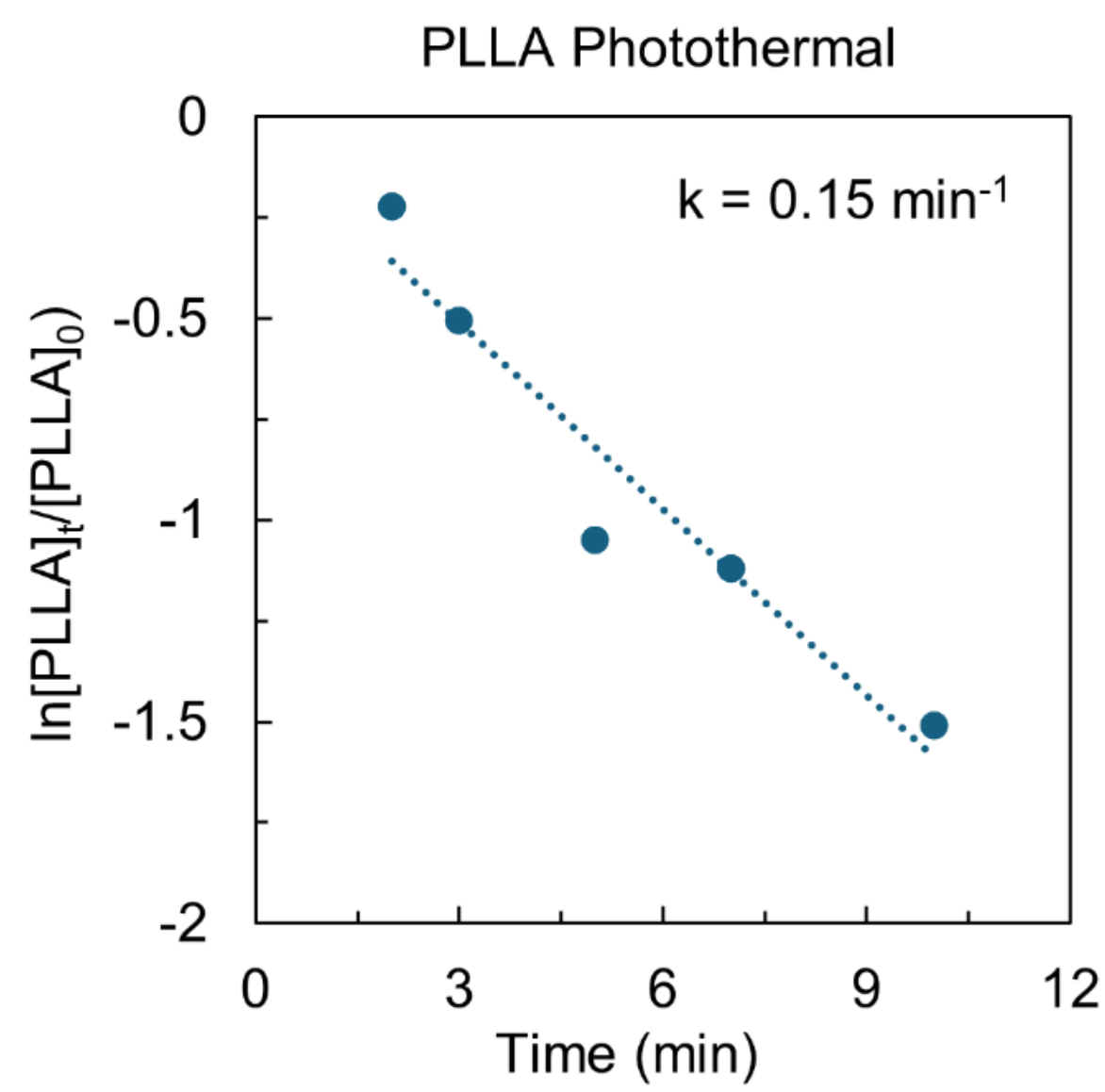

Figure S7. Kinetic plots of photothermal PLLA depolymerization using a 660 nm LED (1.3 W/cm^2) in the presence of $Sn(Oct)_2$ (2.5 mol%) and carbon black (5 wt%).

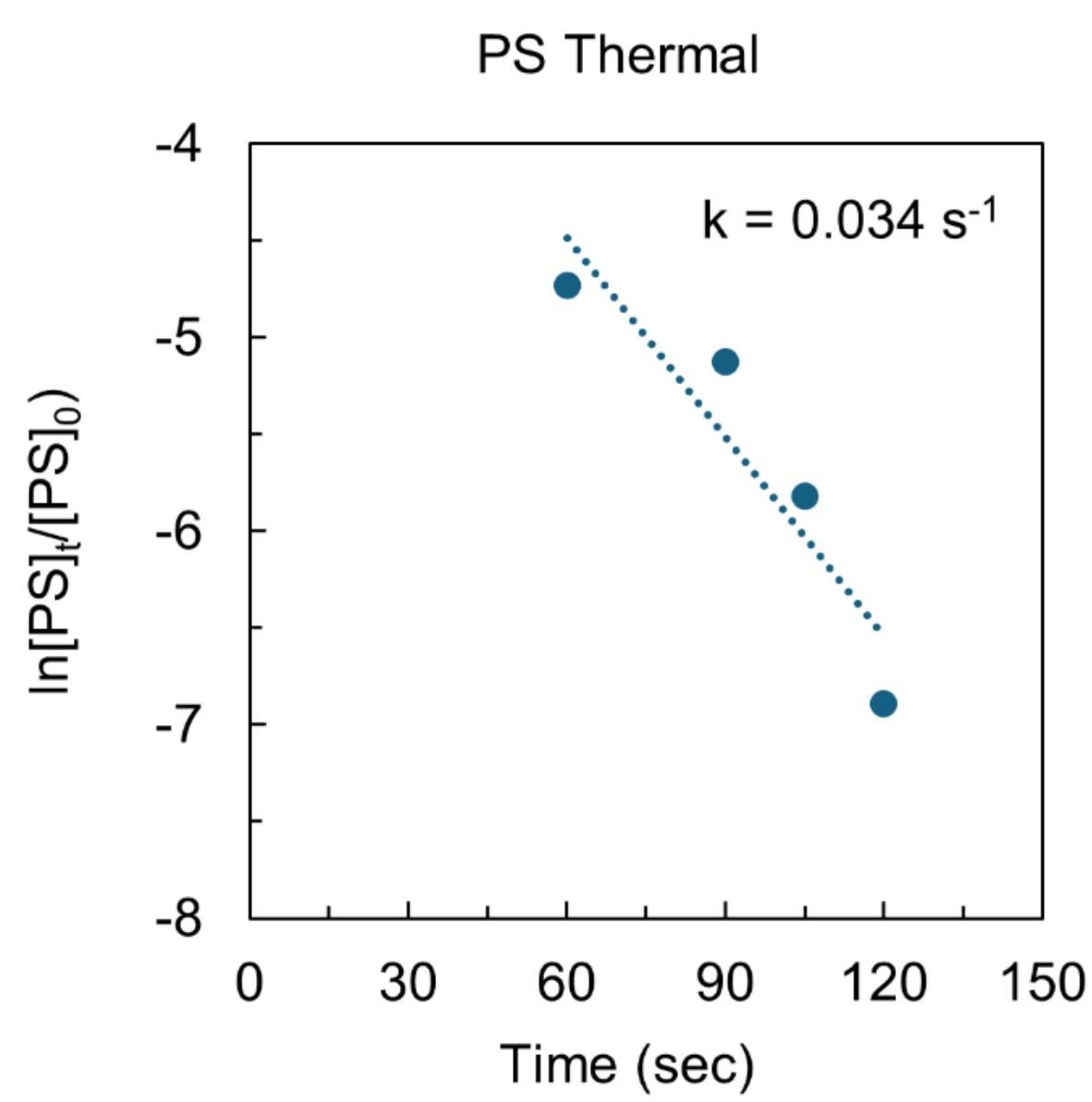

Figure S8. Kinetic plots of PS depolymerization at 400 °C.

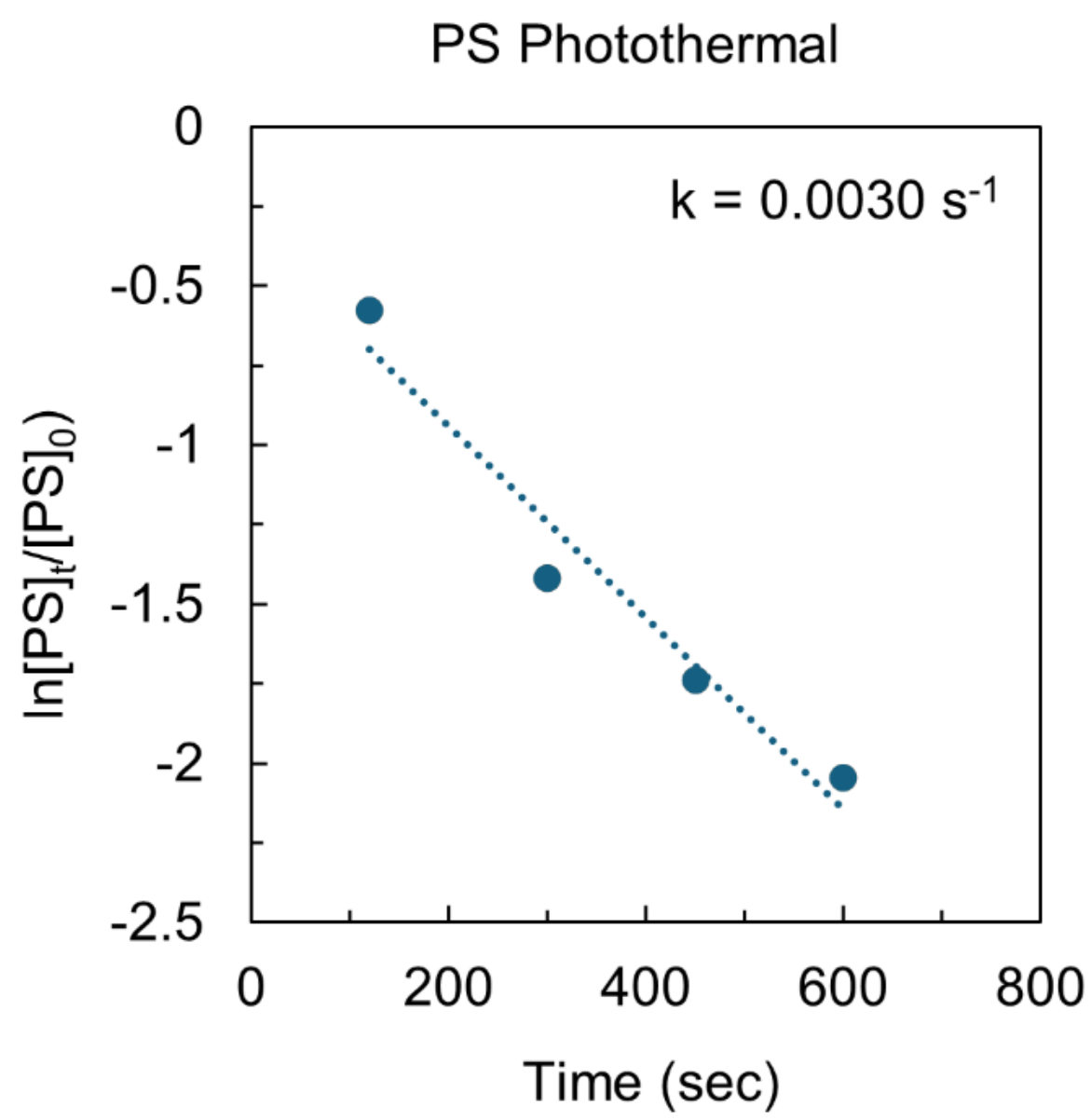

Figure S9. Kinetic plots of photothermal PS depolymerization using a 6000 K white LED (3.0 W/cm^2) in the presence of carbon black (5 wt%).

Table S10. Calculation of Energy Efficiency.

Method	Power of heating equipment	Reaction time (min)	Input electricity	Carbon footprint (g CO_2e)	Styrene yield (%)
Bulk heating at 402 °C	134 W	47 (45 min preheat and equilibrate)	0.10 kW·h	40	60.2
Photothermal conversion	92.9 W	10	0.015 kW·h	6	58.6

Power of heating equipment was measured by power meter. Carbon footprint was estimated based on a value of 0.4 kg CO_2e/kWh.

Characterization of Plastics

Table S11. Characterization of Post-Consumer Plastics Samples (PLLA cup, PET salad container data is from Jang et al).

Polymer	Purity (%)	M_n (kg/mol)[c]	Đ[c]
PLLA (sigma-aldrich)	>99[a]	60.6	2.76
PS (magerial)	>99[a]	71.2	1.94
PLLA cup	>99[a]	84.8	1.87

Black PS coffee cup lid	85.2[a]	64.8	2.52
PET salad container	94.6[b]	N/A[d]	N/A[d]

[a]Measured by ^{1}H NMR spectroscopy in $CDCl_3$ with 1,3,5-trimethoxybenzene as an internal standard. [b]Measured by ^{1}H NMR spectroscopy in trifluoroacetic acid-*d* with 1,3,5-trimethylbenzene as an internal standard. PET was obtained from a salad container. [c]Measured by THF SEC calibrated with PS standard. [d]SEC analysis of the polymer could not be performed due to its low solubility in THF and DMA.

SEC for commercial PLLA, PS, PLLA cup, Black PS cup lid

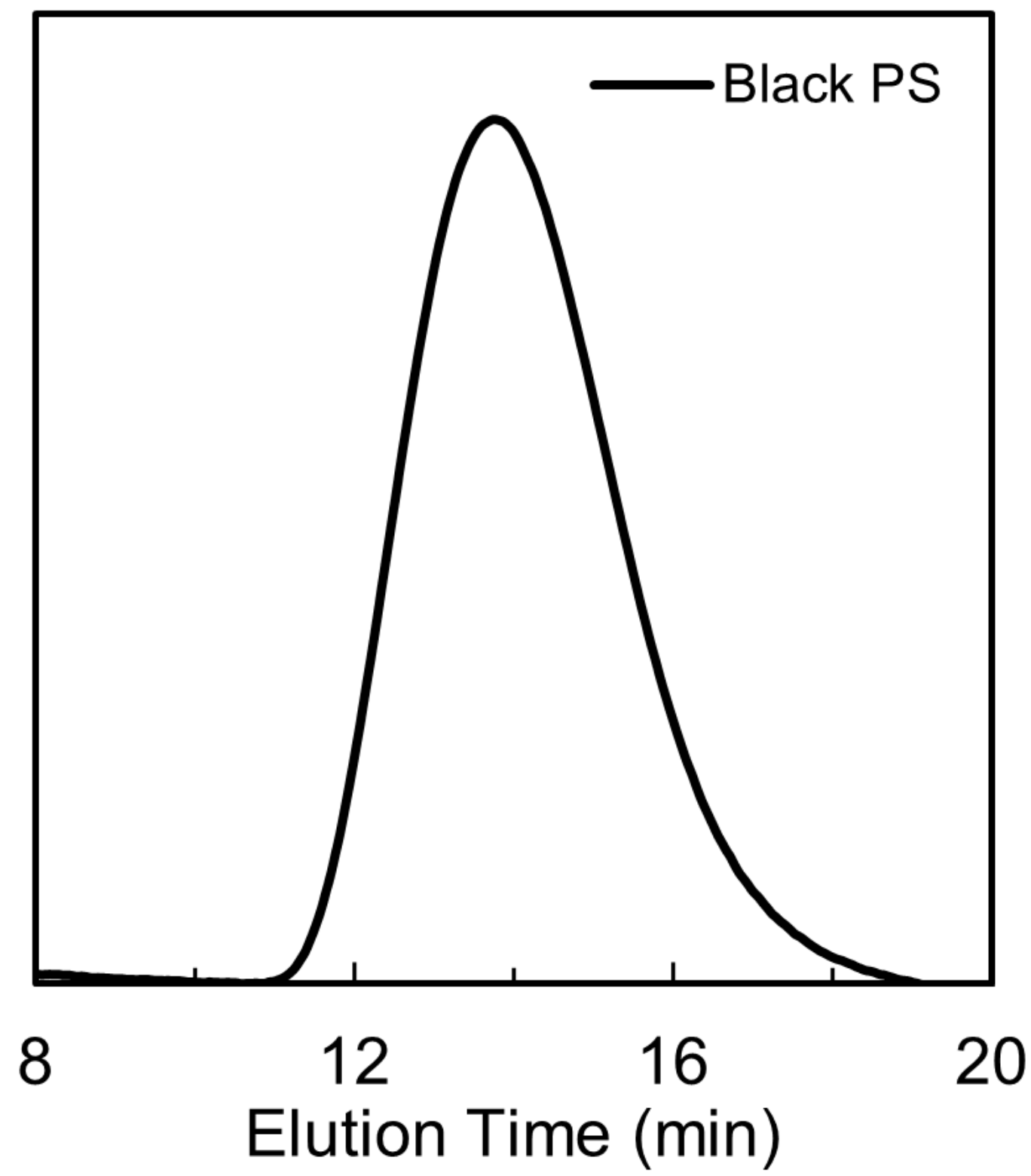

Figure S10. SEC trace for black polystyrene

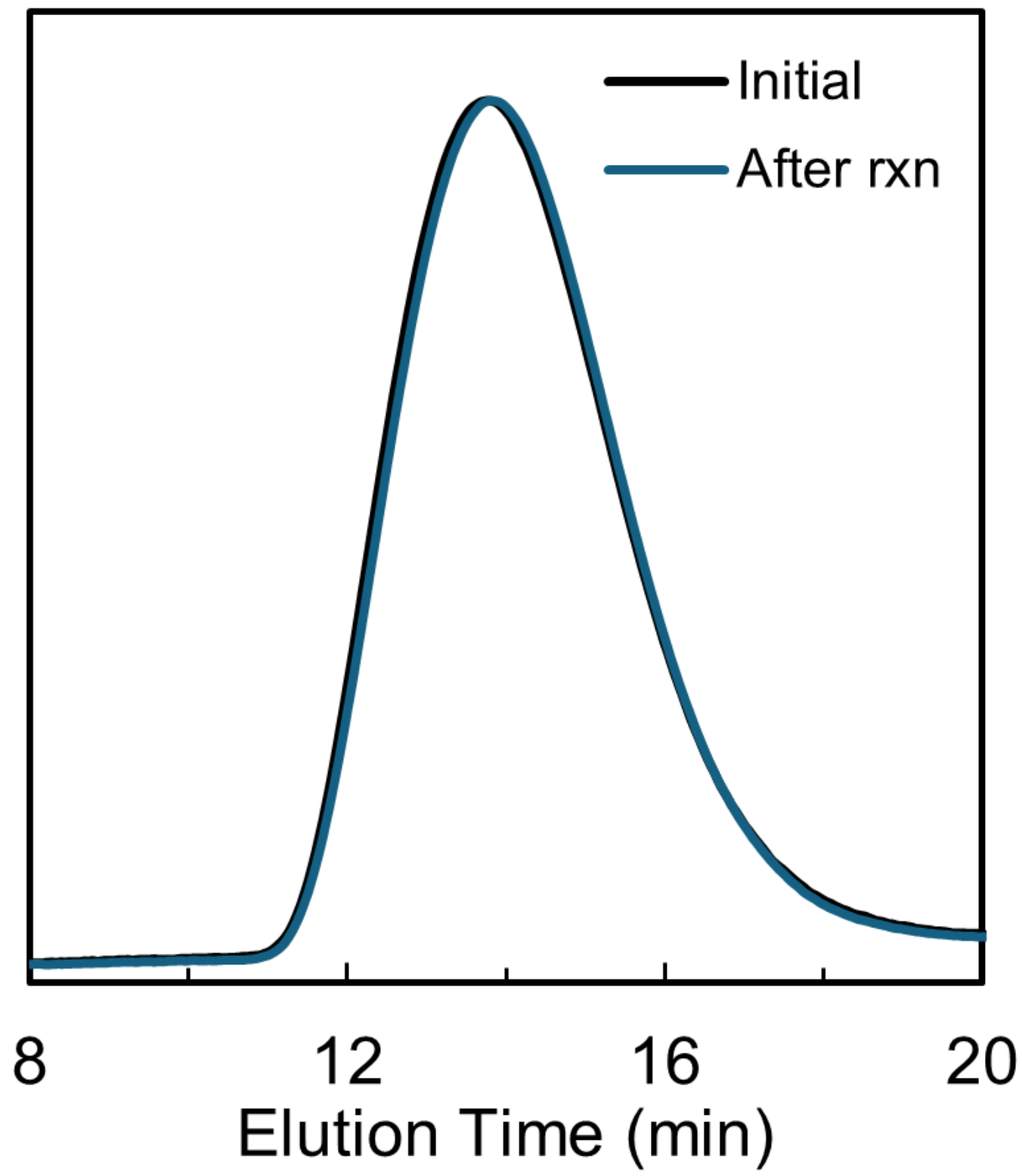

Figure S11. SEC traces for before and after polystyrene for the reaction using 660 nm (1.3 W/cm^2) LED.

^{1}H NMR Spectra

^{1}H NMR Spectrum of Poly(*L*-Lactide)

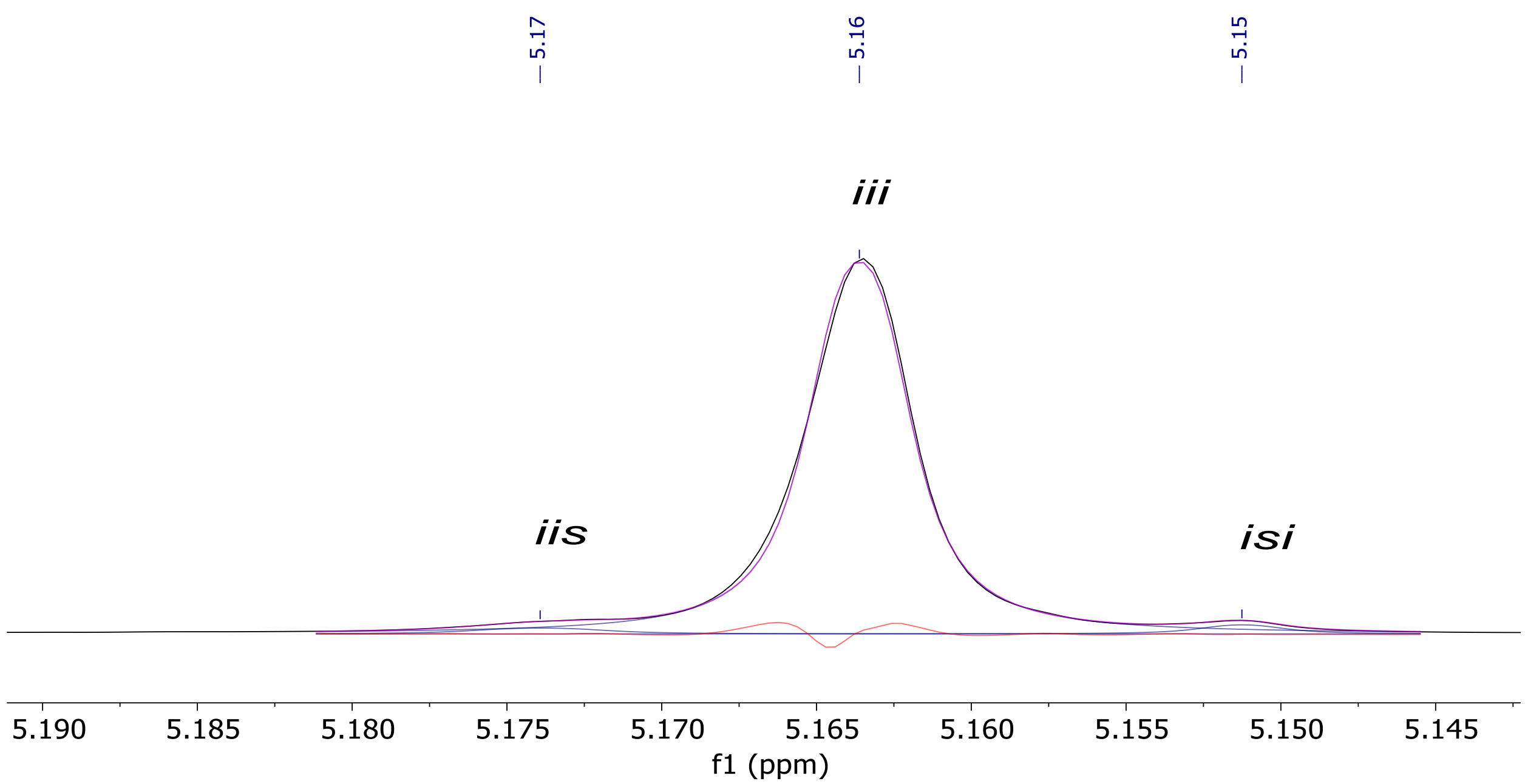

Figure S12. Homonuclear decoupled ^{1}H NMR Spectrum of Poly(*L*-Lactide) (iis/ssi = 2%, iii = 96%, isi = 2% based on the area of the peaks).[2]

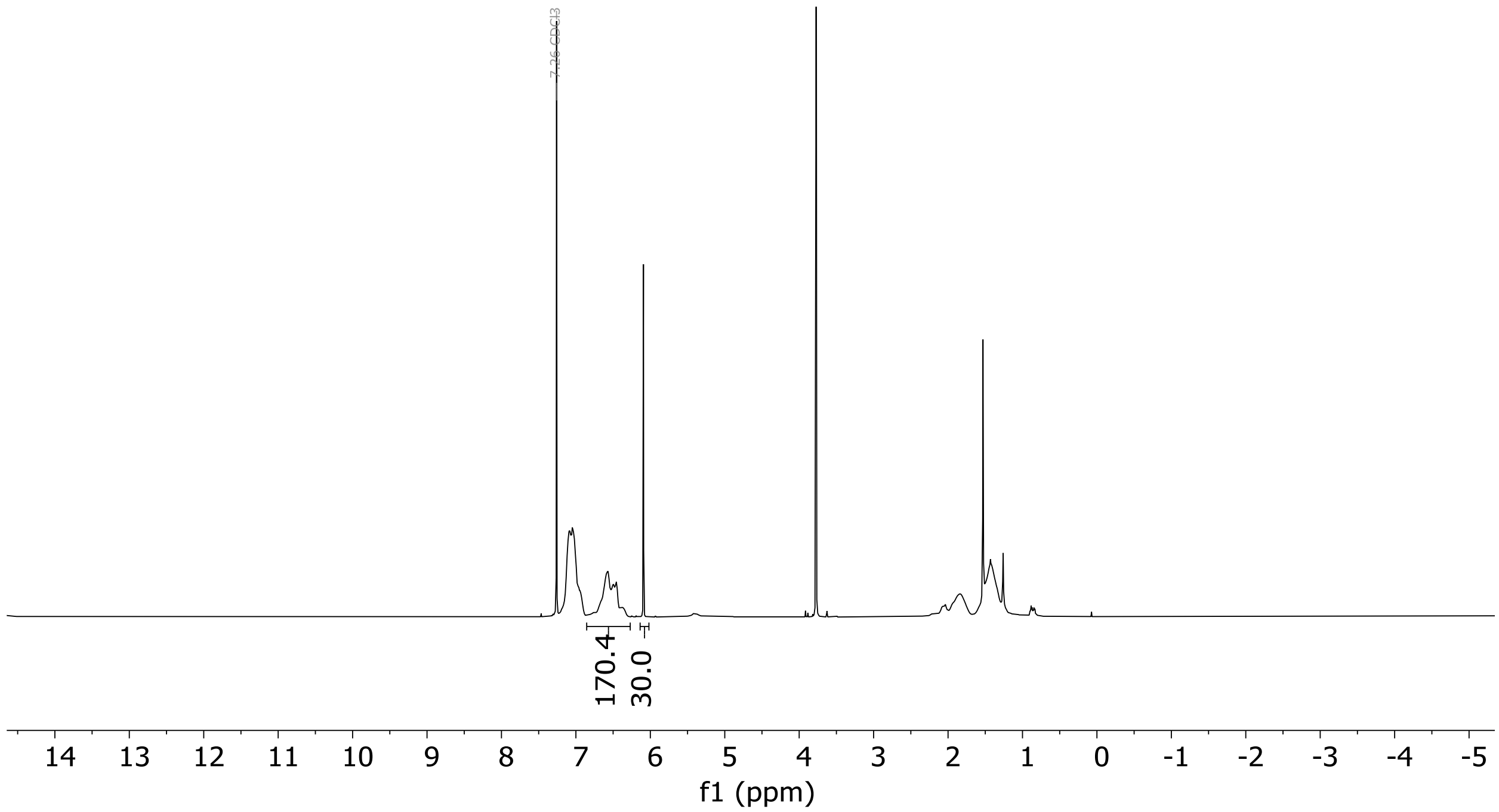

Figure S13. ^{1}H NMR Spectrum of Post-Consumer PS (Black Coffee Cup Lid).

^{1}H NMR Spectra after Depolymerization

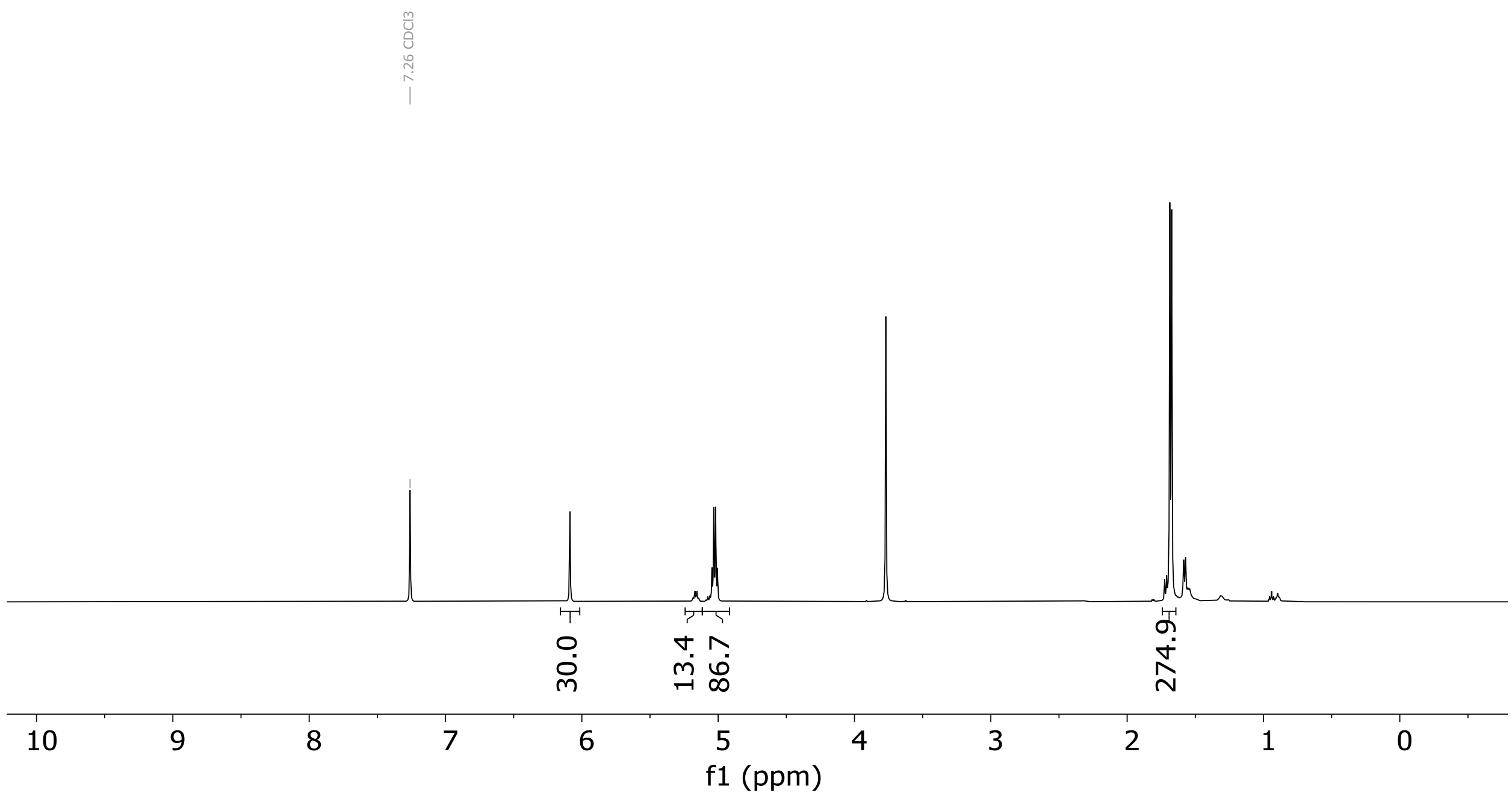

Figure S14. ^{1}H NMR Spectrum after PLLA Depolymerization.

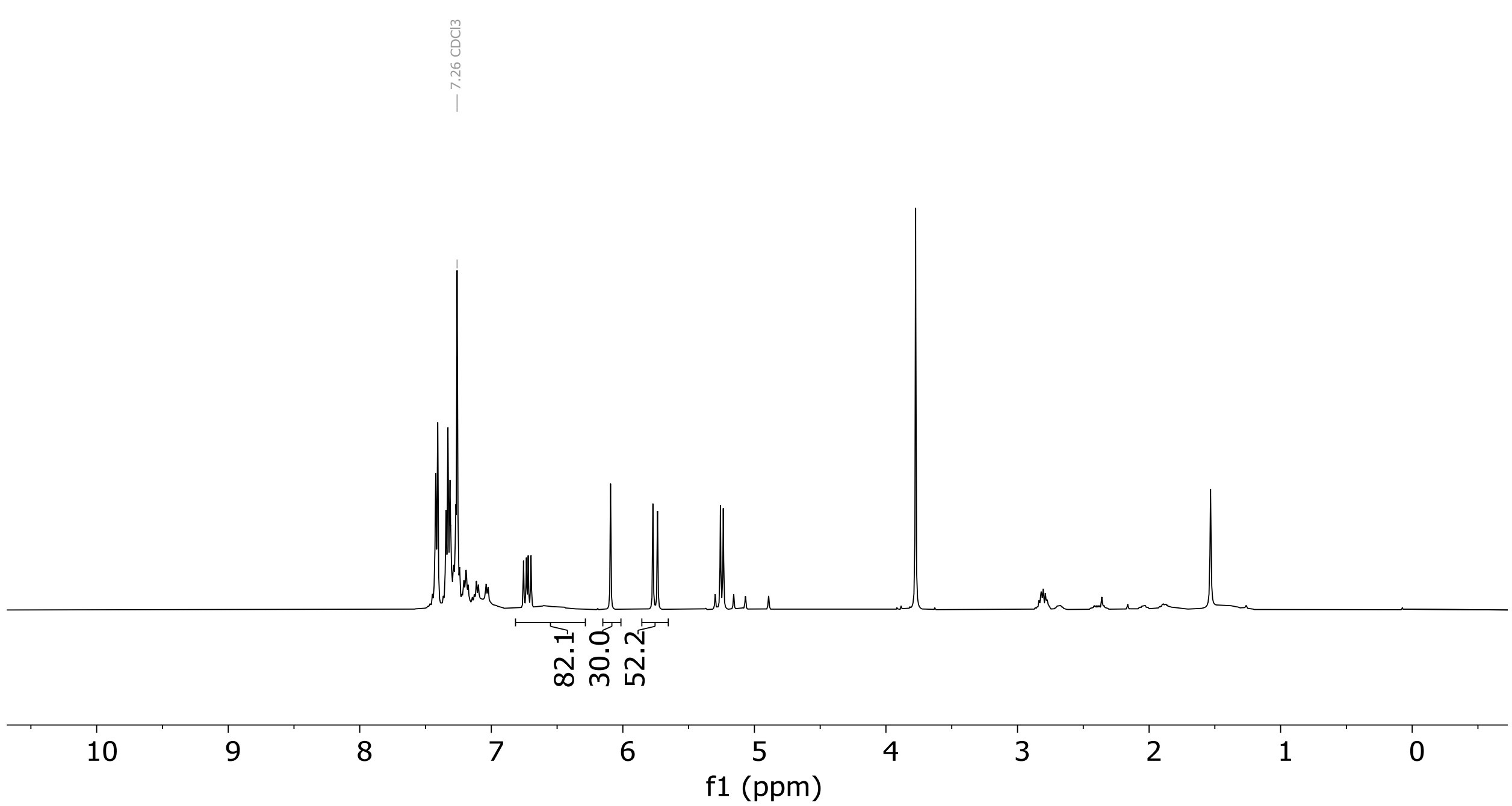

Figure S15. ^{1}H NMR Spectrum after PS Depolymerization.

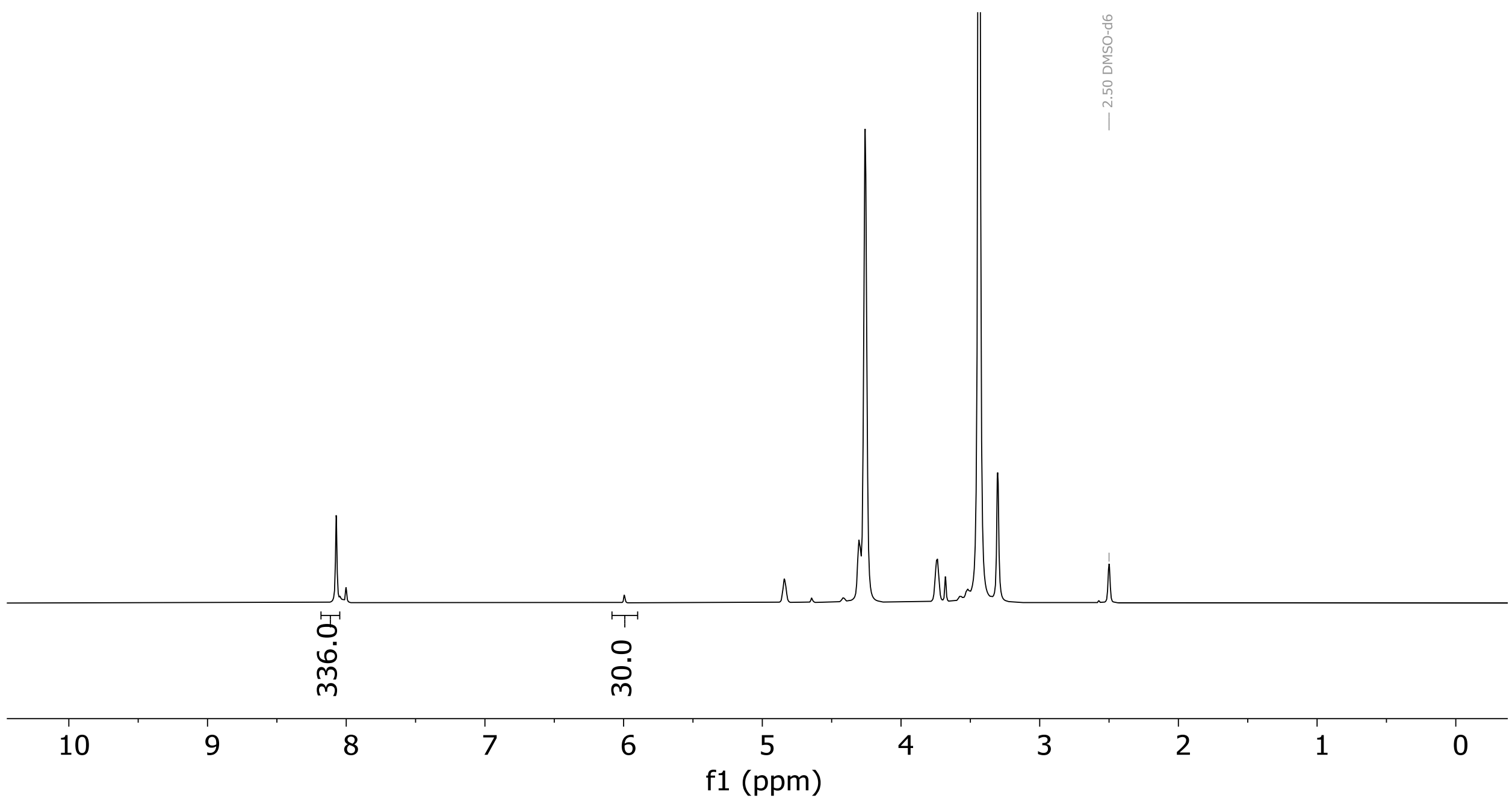

Figure S16. ^{1}H NMR Spectrum after PET Depolymerization.

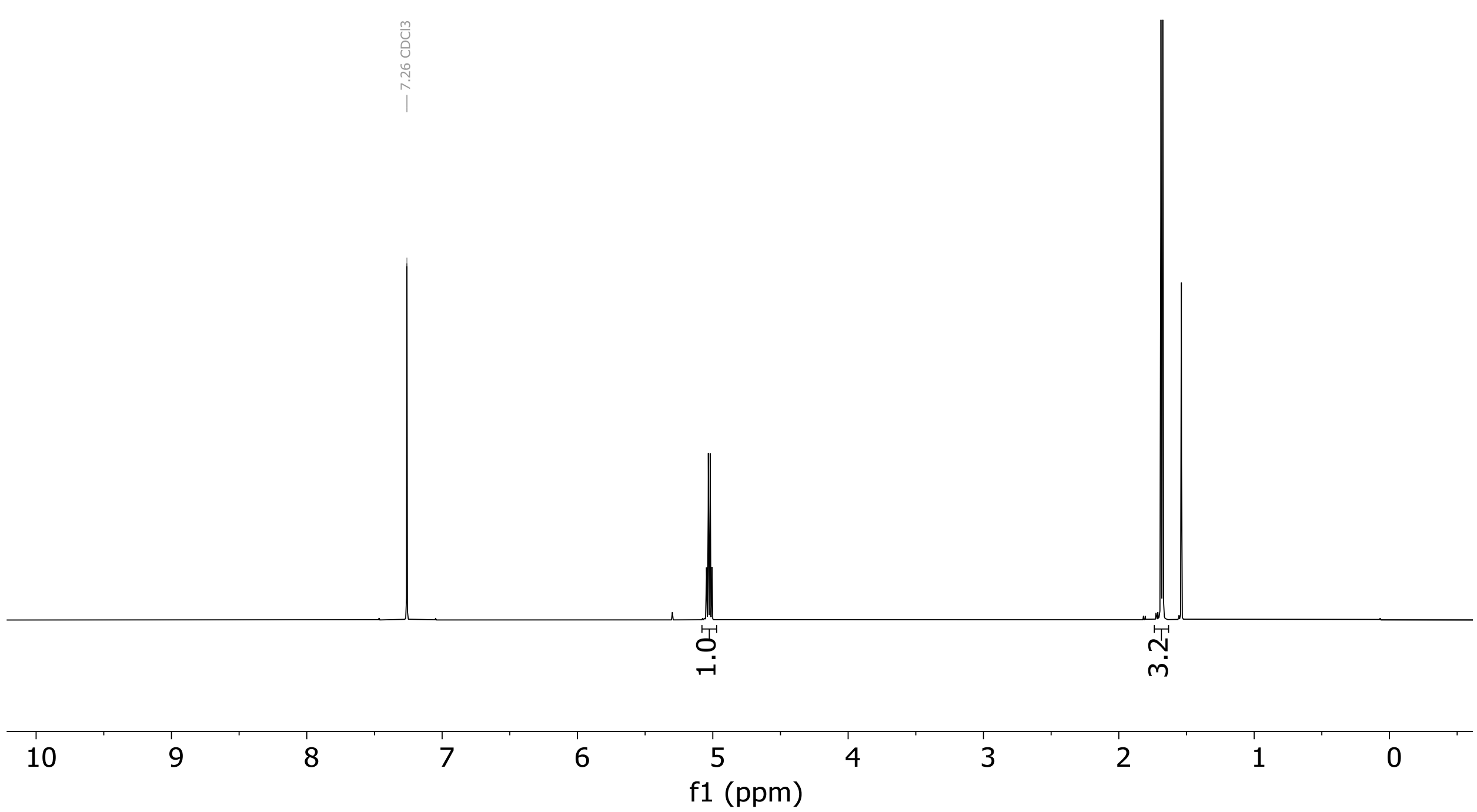

Figure S17. ^{1}H NMR Spectrum after PLLA Depolymerization in PLLA, PET mixture.

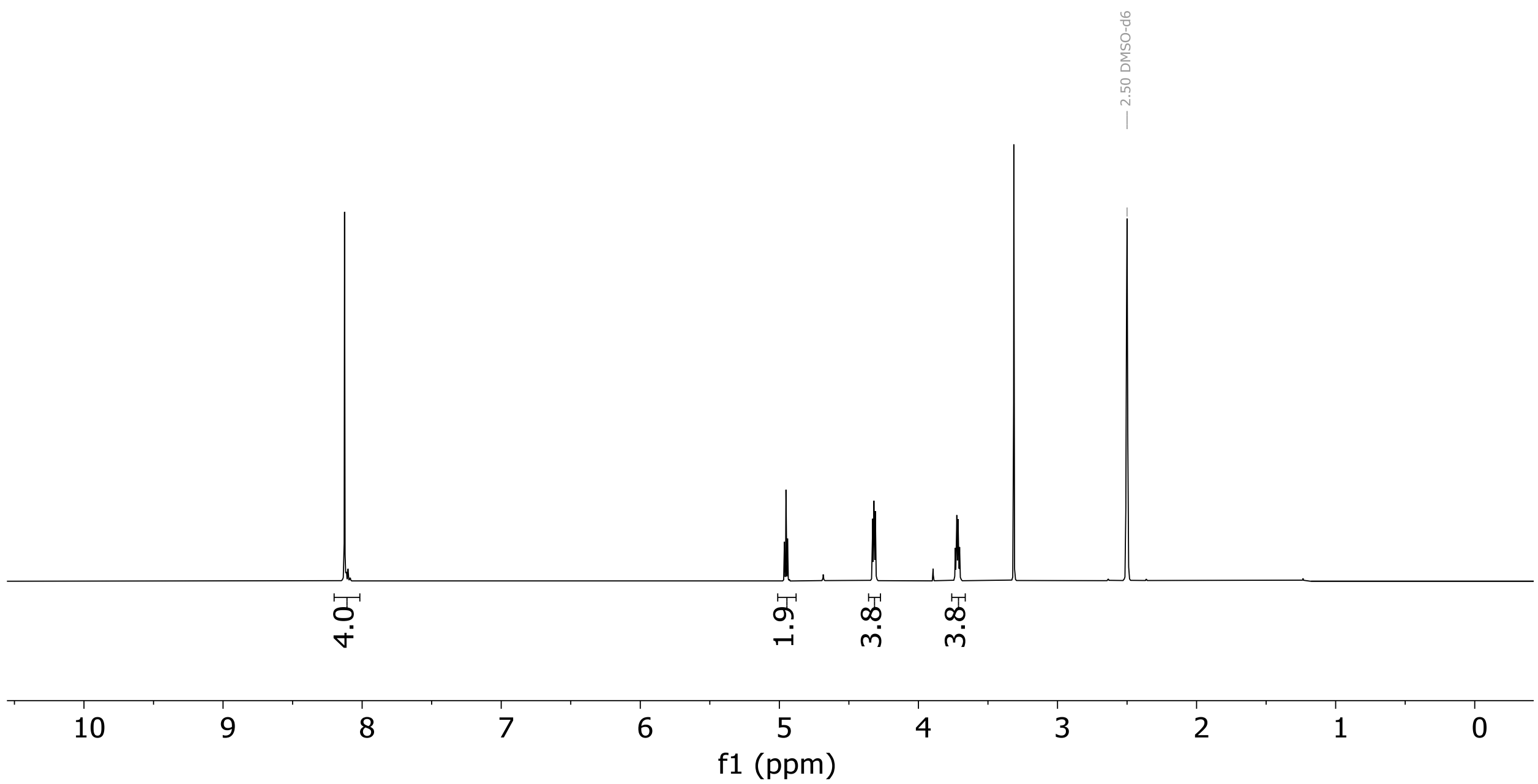

Figure S18. ^{1}H NMR Spectrum after PET Depolymerization in PLLA, PET mixture.

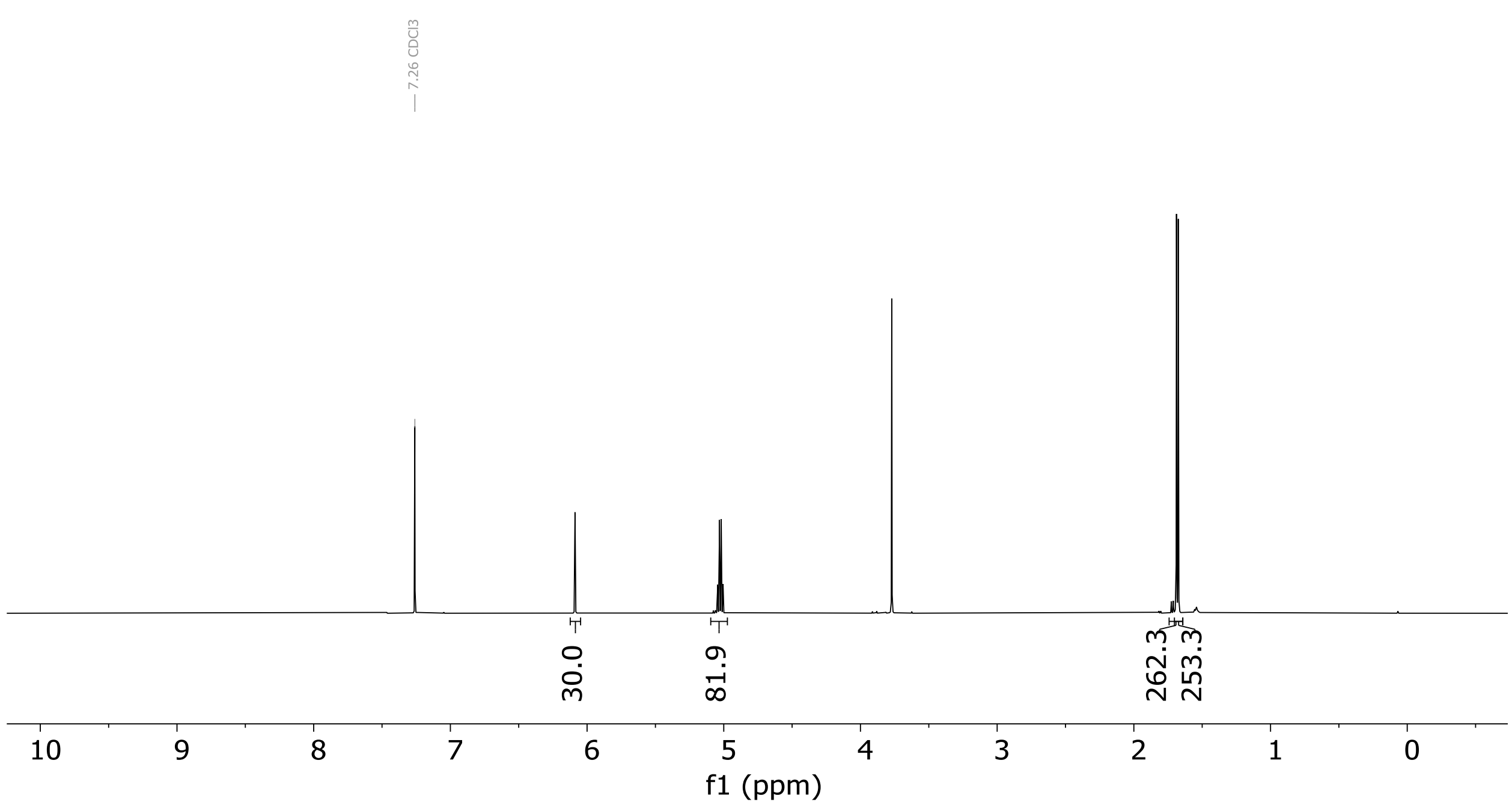

Figure S19. ^{1}H NMR Spectrum of short path after PLLA Depolymerization in PLLA, PS, PET mixture (The yield for Figure 4 was calculated including second wash of short path and flask).

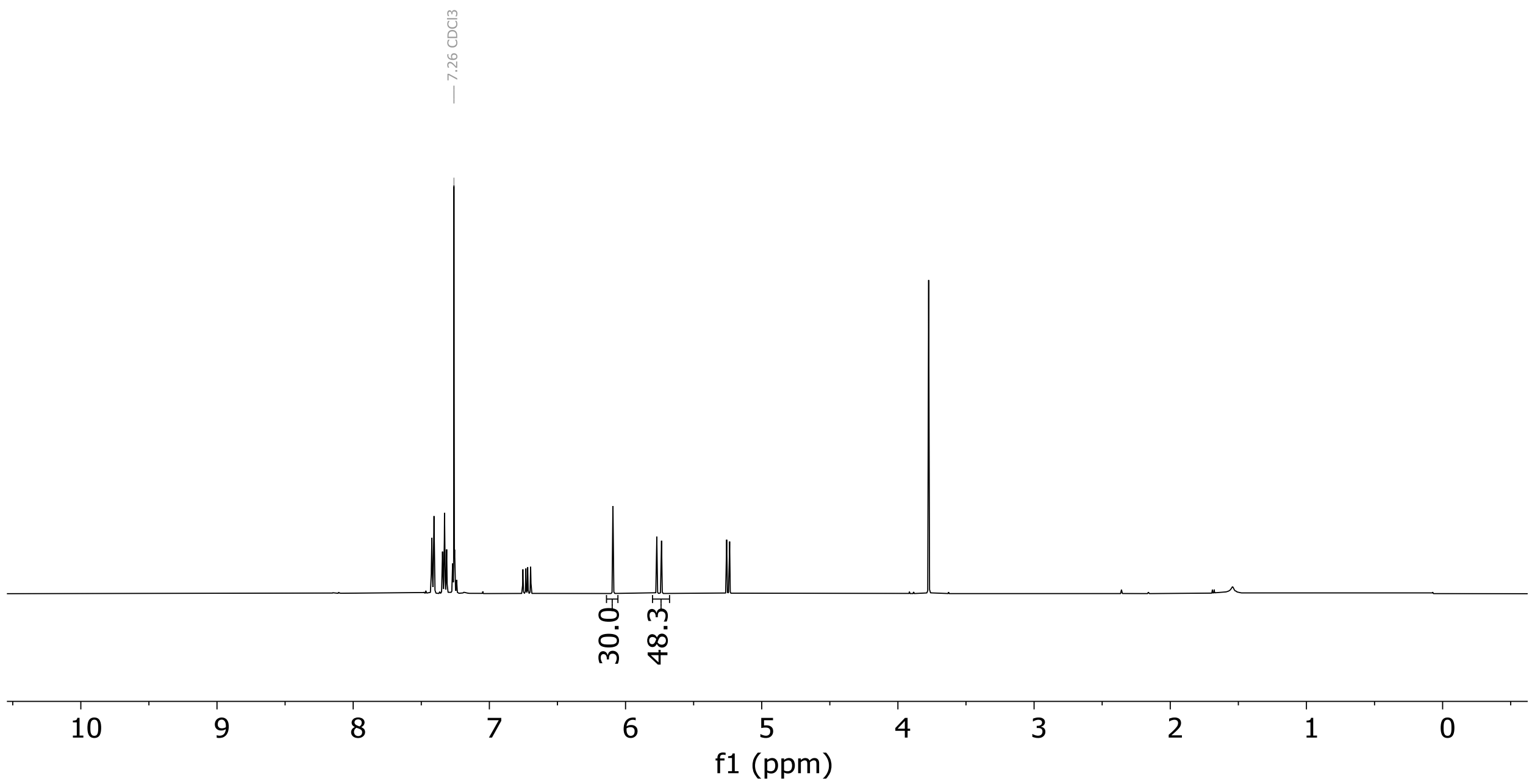

Figure S20. ^{1}H NMR Spectrum after PS Depolymerization in PLLA, PS, PET mixture.

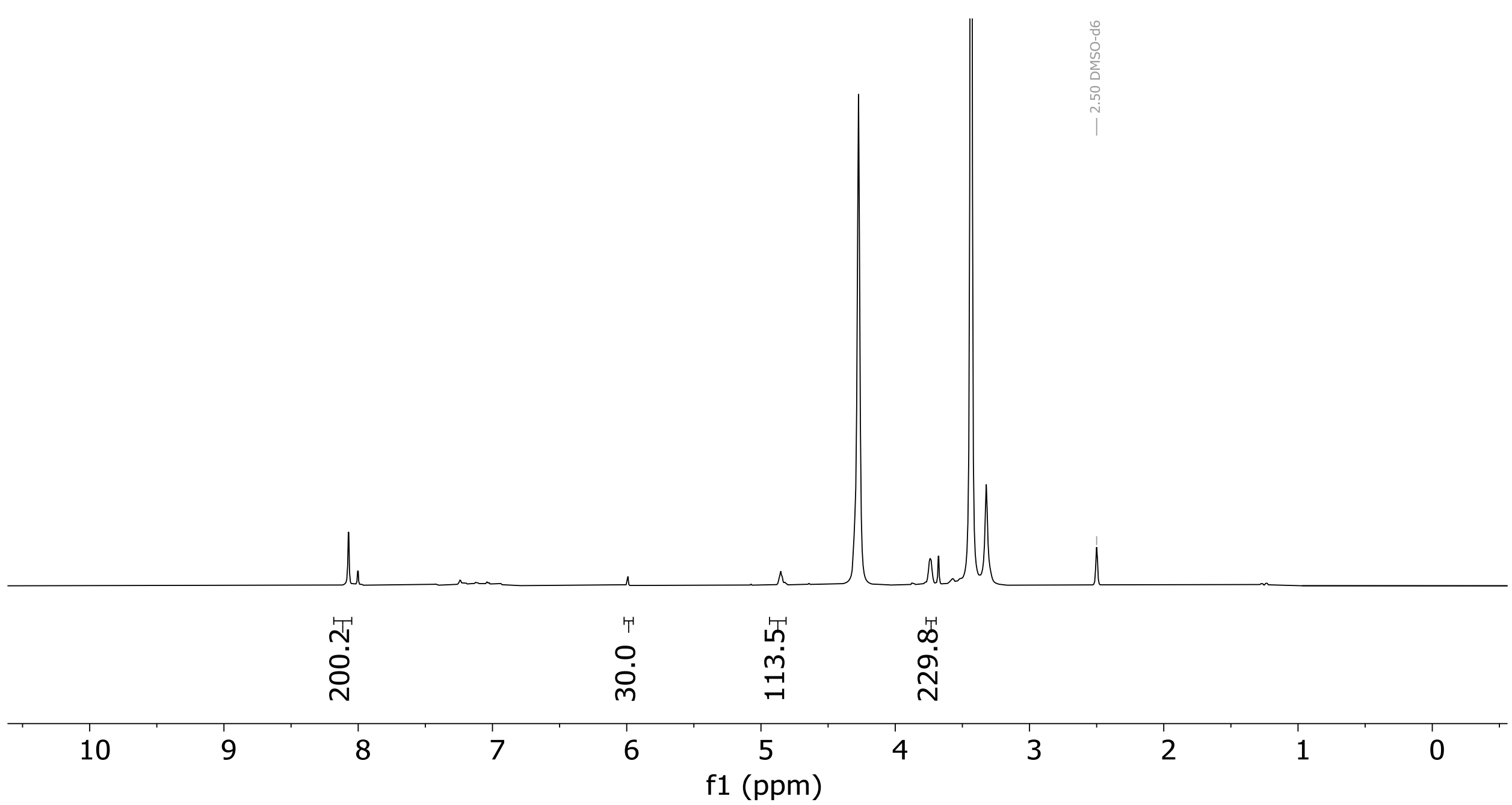

Figure S21. ^{1}H NMR Spectrum after PET Depolymerization in PLLA, PS, PET mixture.

References

(1) Fortenbaugh, R. J.; Lear, B. J. On-demand curing of polydimethylsiloxane (PDMS) using the photothermal effect of gold nanoparticle. *Nanoscale*, **2017**, *9*, 8555.

(2) McGuire, T. M.; Buchard, A.; Williams, C. Chemical Recycling of Commercial Poly(L -Lactic Acid) to L -Lactide Using a High-Performance Sn(II)/Alcohol Catalyst System. *J. Am. Chem. Soc.* **2023**, *145*, 19840.

(3) Kim, S.-S.; Kim, S. Pyrolysis Characteristics of Polystyrene and Polypropylene in a Stirred Batch Reactor. *Chemical Engineering Journal* **2004**, *98*, 53.
